# A lipid-anchored neurokinin 1 receptor antagonist prolongs pain relief by a three-pronged mechanism of action targeting the receptor at the plasma membrane and in endosomes

**DOI:** 10.1016/j.jbc.2021.100345

**Published:** 2021-01-28

**Authors:** Quynh N. Mai, Priyank Shenoy, Tim Quach, Jeffri S. Retamal, Arisbel B. Gondin, Holly R. Yeatman, Luigi Aurelio, Joshua W. Conner, Daniel P. Poole, Meritxell Canals, Cameron J. Nowell, Bim Graham, Thomas P. Davis, Stephen J. Briddon, Stephen J. Hill, Christopher J.H. Porter, Nigel W. Bunnett, Michelle L. Halls, Nicholas A. Veldhuis

**Affiliations:** 1 Drug Discovery Biology Theme, Monash Institute of Pharmaceutical Sciences, Monash University, Parkville, Victoria, Australia; 2 Drug Delivery, Disposition and Dynamics Theme, Monash Institute of Pharmaceutical Sciences, Monash University, Parkville, Victoria, Australia; 3 Australian Research Council Centre of Excellence in Convergent Bio-Nano Science and Technology, Monash Institute of Pharmaceutical Sciences, Monash University, Parkville, Victoria, Australia; 4 Medicinal Chemistry Theme, Monash Institute of Pharmaceutical Sciences, Monash University, Parkville, Victoria, Australia; 5 Division of Physiology, Pharmacology and Neuroscience, School of Life Sciences, The University of Nottingham Medical School, Nottingham, UK; 6 Centre of Membrane Proteins and Receptors, Universities of Birmingham and Nottingham, the Midlands, UK; 7 Australian Institute for Bioengineering and Nanotechnology, University of Queensland, Brisbane, Queensland, Australia; 8 Department of Pharmacology and Therapeutics, The University of Melbourne, Parkville, Victoria, Australia; 9 Department of Molecular Pathobiology, New York University College of Dentistry, New York, New York, USA

**Keywords:** drug delivery, endosome, G-protein-coupled receptor, cell signaling, pain, lipid conjugation, tachykinin, AC, adenylyl cyclase, BACE-1, β-site amyloid precursor protein cleaving enzyme 1, BRET, bioluminescence resonance energy transfer, cAMP, cyclic adenosine monophosphate, Chol, biotin conjugated to cholestanol *via* a PEG linker, CFP, cyan fluorescent protein, Cy5, cyanine 5, Cy5-Chol, cyanine 5 with cholestanol linked via PEG, Cy5-OEt, cyanine 5 with an ethyl ester linked via PEG, cytoCKAR, cytosolic C kinase activity reporter FRET biosensor, cytoEpac2, cytosolic Epac2-camps FRET biosensor, DAG, diacylglycerol, DMEM, Dulbecco’s modified Eagle’s medium, EGFR, epidermal growth factor receptor, ERK, extracellular signal regulated kinase (mitogen activated protein kinase), FBS, fetal bovine serum, FCS, fluorescence correlation spectroscopy, GPCR, G protein-coupled receptor, InsP_3_, inositol trisphosphate, NK_1_R, neurokinin 1 receptor, OEt, ethyl ester, PKA, protein kinase A, PKC, protein kinase C, pmEpac2, plasma membrane localized Epac2-camps FRET biosensor, RLuc8, Renilla luciferase, SP, substance P, Span, Spantide I, Span-Chol, Spantide I conjugated to cholestanol via PEG linker, TAMRA, tetramethylrhodamine, YFP, yellow fluorescent protein

## Abstract

G-protein-coupled receptors (GPCRs) are traditionally known for signaling at the plasma membrane, but they can also signal from endosomes after internalization to control important pathophysiological processes. In spinal neurons, sustained endosomal signaling of the neurokinin 1 receptor (NK_1_R) mediates nociception, as demonstrated in models of acute and neuropathic pain. An NK_1_R antagonist, Spantide I (Span), conjugated to cholestanol (Span-Chol), accumulates in endosomes, inhibits endosomal NK_1_R signaling, and causes prolonged antinociception. However, the extent to which the Chol-anchor influences long-term location and activity is poorly understood. Herein, we used fluorescent correlation spectroscopy and targeted biosensors to characterize Span-Chol over time. The Chol-anchor increased local concentration of probe at the plasma membrane. Over time we observed an increase in NK_1_R-binding affinity and more potent inhibition of NK_1_R-mediated calcium signaling. Span-Chol, but not Span, caused a persistent decrease in NK_1_R recruitment of β-arrestin and receptor internalization to early endosomes. Using targeted biosensors, we mapped the relative inhibition of NK_1_R signaling as the receptor moved into the cell. Span selectively inhibited cell surface signaling, whereas Span-Chol partitioned into endosomal membranes and blocked endosomal signaling. In a preclinical model of pain, Span-Chol caused prolonged antinociception (>9 h), which is attributable to a three-pronged mechanism of action: increased local concentration at membranes, a prolonged decrease in NK_1_R endocytosis, and persistent inhibition of signaling from endosomes. Identifying the mechanisms that contribute to the increased preclinical efficacy of lipid-anchored NK_1_R antagonists is an important step toward understanding how we can effectively target intracellular GPCRs in disease

G-protein-coupled receptors (GPCRs) are tractable therapeutic targets because they have druggable sites on the cell surface and control most pathophysiological processes (1). However, many GPCRs can also signal from intracellular compartments, including endosomes, the Golgi, mitochondria, and the nucleus (2, 3, 4, 5). These intracellular signals dictate physiological responses that are distinct from those that emanate from signaling at the plasma membrane (5, 6, 7, 8, 9, 10). Drug discovery efforts typically target GPCRs at the cell surface, and as a consequence, many drugs targeting GPCRs are not designed to cross the plasma membrane. This inability to effectively engage intracellular GPCRs might explain why some drugs with high efficacy in cell-based assays of plasma membrane signaling fail in clinical trials.

For the GPCR for substance P (SP), the neurokinin 1 receptor (NK_1_R), multiple NK_1_R antagonists have failed in clinical trials of chronic neurological diseases, including pain (11, 12, 13). Activation of the NK_1_R causes two spatially and temporally distinct rounds of signaling (Fig. S1). At the cell surface, SP-bound NK_1_R rapidly activates Gα_q_ G proteins to increase Ca^2+^ mobilization, protein kinase C (PKC) activity, and cyclic adenosine monophosphate (cAMP) formation in the vicinity of the plasma membrane (5, 14). Stimulation of the NK_1_R also leads to transactivation of the epidermal growth factor receptor (EGFR) to increase extracellular signal-regulated kinase (ERK) activity in the cytoplasm. These signals are all relatively short-lived (<15 min) (14). During this time, GPCR kinases rapidly phosphorylate the NK_1_R leading to association with β-arrestins and receptor endocytosis to early endosomes (<2 min) (5). Within endosomes, the SP-NK_1_R complex continues to signal causing increased PKC activity and cAMP in the cytosol and increased ERK activity within the nucleus (5, 14). These signals from the endosomally localized receptor are longer-lived (>20 min). It is these sustained signals from the intracellular NK_1_R that mediate persistent excitation of spinal neurons and sustained central pain transmission (7, 14, 15).

Ligands can have spatially specific or “location biased” pharmacological actions in cells (16). We have previously assessed the potential for drug delivery strategies to locally deliver NK_1_R antagonists to endosomes. This includes pH-responsive nanoparticles that deliver and release the NK_1_R antagonist aprepitant directly into the endosomes (17) and lipid-anchored NK_1_R antagonists that accumulate in endosomal membranes (5). Both of these approaches improved drug efficacy in preclinical models of pain (2–5-fold more effective antinociception, 2–4-fold longer duration of action compared with free drug) (5, 17). The localized delivery of an NK_1_R antagonist to endosomes using nanoparticles is a selective approach that bypasses any effects on receptors at the cell surface. In contrast, lipid-anchored NK_1_R antagonists first partition into the plasma membrane, before they are trafficked to endosomes (5). It is therefore possible that lipid-anchored antagonists also affect the signaling and trafficking of plasma membrane-localized NK_1_R, in addition to their later antagonism of endosomal receptors. This dual antagonism—initial blockade of plasma membrane receptors during partitioning into the plasma membrane and then prolonged blockade of the pathophysiologically relevant signal from endosomes—could enhance therapeutic efficacy.

In the current investigation, we used live cell imaging and biophysical approaches to assess NK_1_R signaling and trafficking in subcellular compartments, in conjunction with behavioral assays of nociception to investigate the mechanisms by which a cholestanol-anchored antagonist, Spantide I (Span-Chol), inhibits endosomal signaling. We used a cholestanol-anchored fluorescent probe (Cy5-Chol) to model the lipid-dependent translocation of the antagonist. We observed that the lipid anchor allows an initial enrichment of probe concentration at the plasma membrane, which correlates with an increased antagonist potency at proximal signaling pathways (*i.e.*, Ca^2+^ mobilization). The lipid-anchored antagonist also inhibits cell surface NK_1_R-β-arrestin recruitment and NK_1_R endocytosis. Over time, Cy5-Chol travels from the plasma membrane to early and late endosomes. This movement deeper into the endosomal network correlates with inhibition of endosomal-selective NK_1_R signaling pathways (5) by Span-Chol, including sustained cytosolic cAMP and cytosolic PKC activity. Consistent with these findings, the lipid-anchored antagonist has long-lasting antinociceptive actions in preclinical models of pain (>9 h).

We find that lipid anchors increase the local membrane concentration of GPCR antagonists, cause inhibition of receptor trafficking from the plasma membrane, and prolong the inhibition of signaling from endosomes. This three-pronged mechanism allows lipid-anchored antagonists to very effectively target endosomally derived GPCR signaling pathways of pathophysiological importance.

## Results

### Lipid anchors increase the available concentration of drug at the cell surface

Inspired by prior studies using lipid–drug conjugates (18, 19), we previously synthesized a series of lipid-anchored probes comprising the sterol cholestanol as a lipid conjugate for anchoring a cargo to membranes *via* a flexible polyethylene glycol linker (PEG4-PEG3-PEG4) (5). For the cargo we used Cyanine 5 (Cy5), to generate a fluorescent reporter of lipid-anchor location (Cy5-Chol), or the NK_1_R antagonist, Spantide I, to generate a lipid-anchored antagonist (Span-Chol) (Fig. S2). We also generated control probes including a nonlipidated fluorescent probe (ethyl-ester group, PEG linker, Cy5; Cy5-OEt) and a lipid anchor control probe (cholestanol group, PEG linker, biotin; Chol).

**Figure 1:**
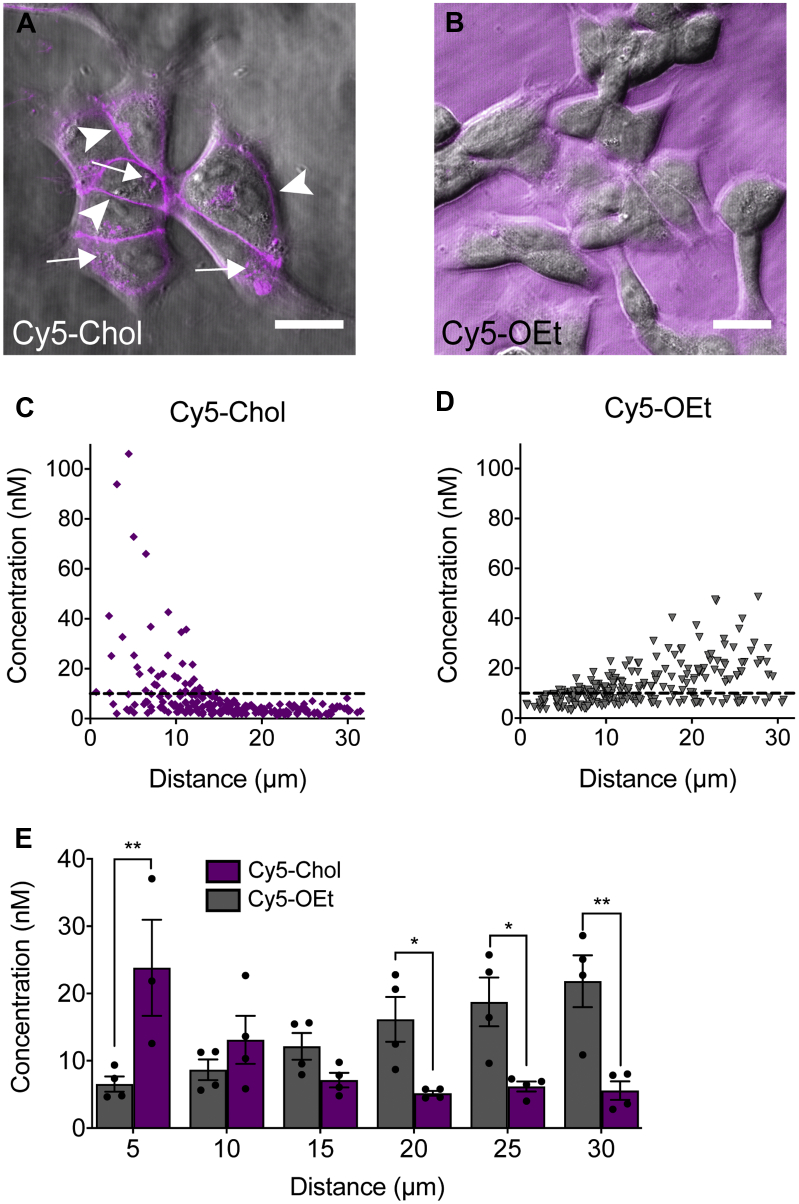
**A cholestanol lipid anchor increases the concentration of Cy5 immediately above the plasma membrane.***A* and *B*, Confocal images of HEK293 cells after incubation with 1 μM Cy5-Chol (*A*) or Cy5-OEt (*B*). *Arrows* indicate intracellular Cy5 fluorescence, and *arrow heads* indicate Cy5 fluorescence at the plasma membrane. Scale bar, 20 μm. *C* and *D*, the concentration of 10 nM solution of Cy5-Chol (*C*) or Cy5-OEt (*D*) at increasing distances above the plasma membrane of HEK293 cells was calculated using FCS. Data points show the concentrations measured at six distance intervals averaged from 3 to 4 independent experiments. The nominal concentration of the added solution (10 nM) is shown by a *dashed line*. *E*, the concentration of Cy5-Chol and Cy5-OEt binned at increasing 5 μm intervals above the plasma membrane. Bars show the mean, error bars show the standard error of the mean (S.E.M.), and data points show the average concentrations obtained from each individual experiment (n = 4). ∗*p* < 0.05, ∗∗*p* < 0.01 Cy5-OEt vs Cy5-Chol; two-way ANOVA with Sidak’s multiple comparison test.

Fluorescence correlation spectroscopy (FCS) enables measurement of the concentrations of fluorescent molecules within a small defined volume (<0.2 fl) (20, 21). We used this approach to determine the concentration of Cy5 probes (Cy5-Chol or the control, Cy5-OEt) in the extracellular fluid immediately above the plasma membrane and at increasing distances above the cell (30–200 μm). We chose the Cy5 probes as the simplest example of how a lipid anchor could affect the plasma membrane concentration of a cargo, independent of any receptor-dependent effects on ligand distribution (22). Consistent with our previous studies (5), brightfield and fluorescence confocal imaging confirmed that Cy5-Chol rapidly incorporated into the plasma membrane of HEK293 cells (Fig. 1*A*), but Cy5-OEt remained in extracellular fluid (Fig. 1*B*). We then used FCS to quantify the concentration of Cy5 fluorescence at the plasma membrane of cells incubated with a nominal concentration of probe (10 nM). The concentration of Cy5-Chol in the extracellular fluid at 5 μm above the plasma membrane was 23.8 ± 7.1 nM, which decreased more than fourfold to 5.6 ± 1.4 nM at 30 μm (mean ± SEM from n = 4) (Fig. 1, *C* and *E*). In contrast, the measured concentration of Cy5-OEt was 6.5 ± 1.1 nM at 5 μm above the plasma membrane, which increased more than threefold to 21.8 ± 3.8 nM at 200 μm (mean ± SEM from n = 4) (Fig. 1, *D* and *E*). A comparison of probe concentrations at increasing distances (5 μm intervals) above the plasma membrane suggested that there was an enrichment of Cy5-Chol proximal to the plasma membrane, while the Cy5-OEt reporter molecule could freely diffuse through the extracellular fluid (Fig. 1*E*). Therefore, the addition of a lipid anchor results in an enhanced association of a probe with cell membranes. This creates a high local concentration of probe at the cell surface.

### Lipid anchoring increases the affinity and potency of an NK_1_R antagonist

To determine whether the addition of a lipid anchor influences the affinity and potency of an NK_1_R antagonist, we compared unconjugated (“free”) Spantide I (Span) and Span-Chol. A high-content imaging competition binding assay was used to evaluate the capacity of these antagonists to disrupt the binding of SP labeled with fluorescent tetramethylrhodamine (SP-TAMRA) to the NK_1_R stably transfected in HEK293 cells. Cells were analyzed using an established granularity algorithm to provide a measure of total cell binding (includes both cell surface and intracellular) (21, 23). We assessed antagonist affinity at two time points following antagonist addition: 30 min, when FCS data show Cy5-Chol enrichment at the plasma membrane (Fig. 1); and 4 h, when Span-Chol accumulates, and is pharmacologically active, within endosomal compartments (5).

**Figure 2:**
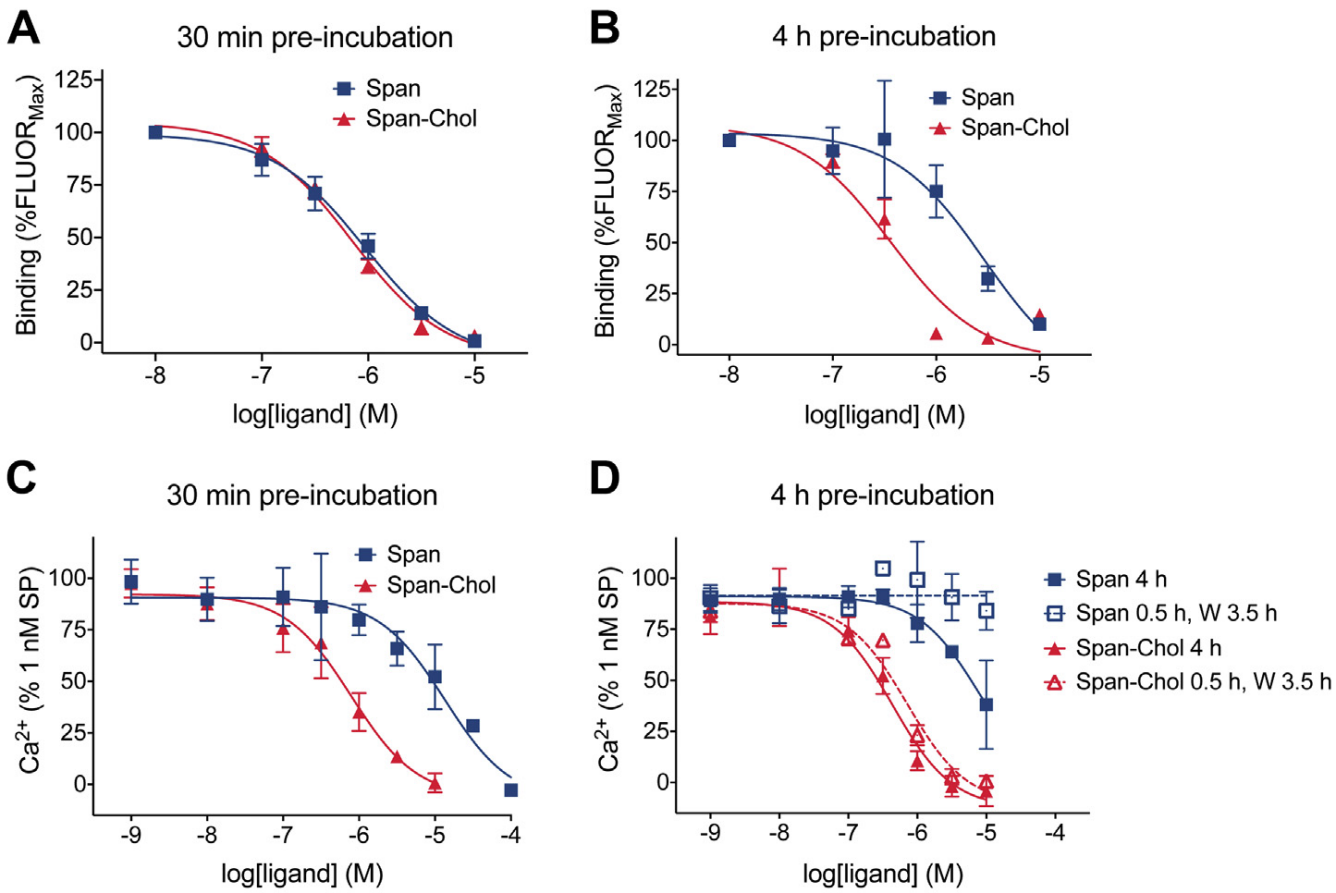
**A cholestanol lipid anchor increases the relative affinity and potency of an NK**_**1**_**R antagonist.***A* and *B*, the affinity of Span compared to Span-Chol was assessed by competition with fluorescent SP-TAMRA in HEK-NK_1_R cells by high-content imaging (n = 5). HEK-NK_1_R cells were preincubated with vehicle (0.1% v/v DMSO; total binding) or increasing concentrations of Span or Span-Chol for a total of 30 min (*A*) or 4 h (*B*) at 37°C prior to addition of 0.5 nM SP-TAMRA. Data are expressed as a percentage of the fluorescent intensity measured in the presence of 10 nM Span or Span-Chol (%FLUOR_Max_). Symbols show means, and error bars S.E.M. of five independent experiments performed in triplicate. *C* and *D*, Calcium transients were measured in HEK-NK_1_R cells in response to 1 nM SP following short (30 min; *C*) or long (4 h; *D*) preincubation with increasing concentrations of Span or Span-Chol (n = 3). Four-h preincubation experiments compared continuous exposure to antagonist (4 h) versus a “pulsed” exposure (0.5 h exposure, wash [W], 3.5 h rest). Symbols show means, and error bars S.E.M. of three independent experiments performed in triplicate.

To assess competition binding after 30 min, HEK-NK_1_R cells were coincubated with an EC_50_ concentration of SP-TAMRA (0.5 nM) and increasing concentrations of Span or Span-Chol and equilibrated for 30 min. The affinity of Span-Chol and Span for NK_1_R was similar with pIC_50_ values of 6.28 ± 0.09 and 5.99 ± 0.13, respectively (Fig. 2*A*). Therefore, peptide modification by attachment of a PEG_12_ linker and cholestanol anchor does not diminish the affinity of Spantide for the NK_1_R.

To assess ligand binding after 4 h, HEK-NK_1_R cells were preincubated with antagonist for 3.5 h, then with SP-TAMRA for a further 30 min (4 h total). The affinity of Span for the NK_1_R was significantly reduced compared with that of Span-Chol (pIC_50_ 5.55 ± 0.17 vs 6.50 ± 0.12, *p* = 0.0018, unpaired *t*-test) (Fig. 2*B*). However, there was no significant change in the relative affinities of Span or Span-Chol for the NK_1_R over time (*p* = 0.1121 and *p* = 0.6378, respectively; one-way ANOVA with Tukey’s multiple comparisons test). This suggests that the addition of a lipid anchor improves the kinetic properties of Span by sustaining its ability to compete with SP-TAMRA at the NK_1_R over a 4 h period. Our previous studies indicated no difference in stability of these ligands in spinal cord membranes (5). We therefore propose that this apparent improvement in affinity of Span-Chol for the NK_1_R is due to the accumulation of Span-Chol in endosomes, allowing Span-Chol to access both plasma membrane and endosomal pools of NK_1_R.

To determine if lipid conjugation influenced the potency of Span, we compared the ability of Span and Span-Chol to inhibit SP-stimulated Ca^2+^ signaling in HEK-NK_1_R cells at different time points after addition. In initial experiments, HEK-NK_1_R cells were preincubated with increasing concentrations of Span or Span-Chol for 30 min, prior to challenge with an EC_80_ concentration of SP (1 nM). Ca^2+^ transients were measured for 90 s poststimulation. Preincubation of cells with Span or Span-Chol caused a concentration-dependent inhibition of Ca^2+^ flux (Fig. 2*C*). A comparison of the pIC_50_ values of Span and Span-Chol (4.87 ± 0.33 and 6.25 ± 0.19, respectively) revealed a significant increase in the potency of the lipidated antagonist (*p* = 0.0112). This is consistent with FCS experiments (Fig. 1) and may be due to the lipid anchoring of the antagonist to the plasma membrane, thereby effectively increasing the local concentration of the antagonist near the receptor even at acute time periods (24, 25).

While lipid-anchored fluorescent probes initially partition into the plasma membrane, they are then quickly trafficked into endosomal compartments (5). As such, the continuous removal of lipidated antagonists from the plasma membrane by constitutive endocytosis could affect the relative potency of Span-Chol compared with soluble Span over time. To assess this possibility, we compared continuous exposure to the antagonists for 4 h to a “pulsed” administration whereby the cells were preincubated with antagonist for 30 min, washed to remove any excess ligand, and then left at 37°C for 3.5 h (4 h total). In both protocols, cells were challenged with 1 nM SP 4 h after the initial antagonist addition. There was no change in the pIC_50_ of the antagonists when the cells were continuously incubated with Span or Span-Chol for 4 h (5.11 ± 0.76 and 6.36 ± 0.17, respectively) compared with the 30 min preincubation (Fig. 2*D*).

After pulsed administration, only Span-Chol retained its ability to antagonize SP-stimulated Ca^2+^ signaling at 4 h (pIC_50_ 6.15 ± 0.11) (Fig. 2*D*). This is likely due to the wash step (after the initial 30 min incubation with antagonist) decreasing the available concentration of free Span in the extracellular fluid. In contrast, the potency of Span-Chol was not lost following the wash, confirming that lipidation causes an increased association of the antagonist with the cell membrane. Notably, the potency of Span-Chol was sustained over 4 h despite the increasing internalization of lipid-anchored probes over time (5). This could indicate a prolonged retention of the lipid-anchored antagonist at the plasma membrane (in addition to internalization into the endosomal network).

Together, these data demonstrate that cholestanol conjugation can enhance the potency and affinity of antagonists by increasing their retention in the plasma membrane and therefore their effective local concentration.

### A lipid-anchored antagonist decreases endocytosis of the activated NK_1_R

Span-Chol has a high local concentration at the cell surface (Fig. 1) and maintains antagonistic activity at cell surface receptors even after 4 h (Fig. 2). It is therefore possible that lipidated antagonists continually act at the plasma membrane to inhibit SP-induced endocytosis of the NK_1_R, which could contribute to their long-lasting therapeutic efficacy. To assess this possibility, we measured the proximity between NK_1_R-RLuc8 and β-arrestin2-YFP, KRas-Venus (marker of the plasma membrane), or Rab5a-Venus (marker of early endosomes) in HEK293 cells using BRET. We compared the effectiveness of Span versus Span-Chol after short (30 min) or prolonged (4 h) incubation. In order to observe any differences between the antagonists that were due to the prolonged retention of Span-Chol at the cell surface, we used the “pulsed” incubation protocol: 30 min antagonist, wash, 3.5 h recovery (4 h total).

**Figure 3:**
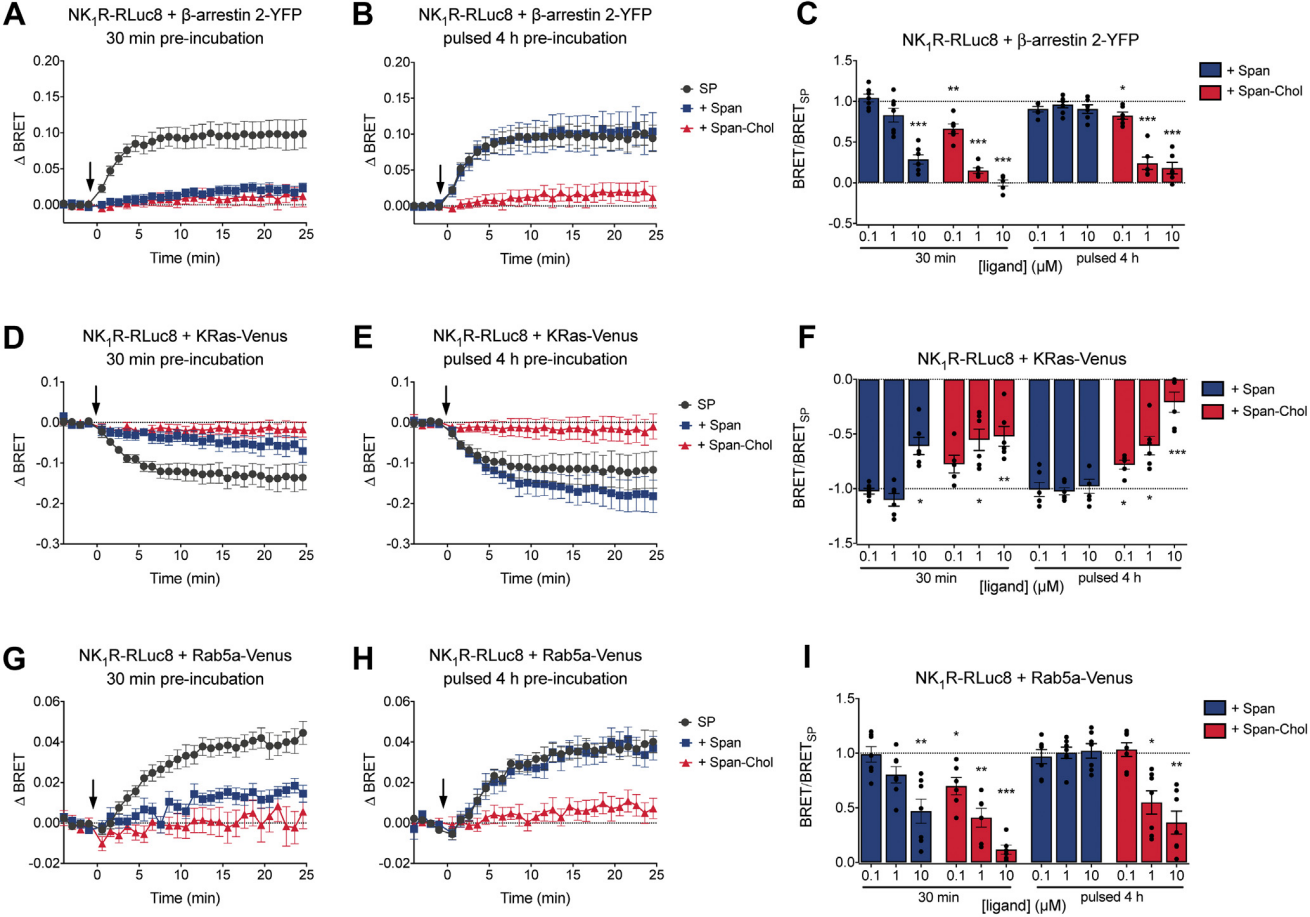
**Span-Chol causes sustained inhibition of NK**_**1**_**R-induced recruitment of β-arrestin and receptor internalization to early endosomes.** The effect of short (30 min) versus long (pulsed 4 h: 30 min treatment, wash, 3.5 h recovery) preincubation with Span or Span-Chol on the NK_1_R-induced recruitment of β-arrestin and receptor internalization to early endosomes was determined using BRET in HEK cells (n = 6). *A–C*, 1 nM SP-induced change in BRET between NK_1_R-Rluc8 and β-arrestin 2-YFP after preincubation with 10 μM Span or Span-Chol for 30 min (*A*) or pulsed 4 h (*B*). *C*, the plateau response calculated from curve fit of the BRET time courses (as per *A* and *B*) after preincubation with 0.1 μM, 1 μM, or 10 μM antagonist, expressed relative to SP alone. *D–F*, 1 nM SP-induced change in BRET between NK_1_R-Rluc8 and KRas-Venus after preincubation with 10 μM Span or Span-Chol for 30 min (*D*) or pulsed 4 h (*E*). *F*, the plateau response calculated from curve fit of the BRET time courses (as per *D* and *E*) after preincubation with 0.1 μM, 1 μM, or 10 μM antagonist, expressed relative to SP alone. *G–I*, 1 nM SP-induced change in BRET between NK_1_R-Rluc8 and Rab5a-Venus after preincubation with 10 μM Span or Span-Chol for 30 min (*G*) or pulsed 4 h (*H*). *I*, the plateau response calculated from curve fit of the BRET time courses (as per *G* and *H*) after preincubation with 0.1 μM, 1 μM, or 10 μM antagonist, expressed relative to SP alone. For time courses, symbols show means and error bars S.E.M; for bar graphs, columns show means, error bars show S.E.M., and symbols show the mean of each individual experiment performed in duplicate. ∗*p* < 0.05, ∗∗*p* < 0.01 and ∗∗∗*p* < 0.001 versus SP alone, two-way ANOVA with Dunnett’s multiple comparisons test.

In control cells, SP induced an increase in NK_1_R-RLuc8/β-arrestin2-YFP BRET, consistent with β-arrestin2 recruitment to NK_1_R (Fig. 3, *A–C*). After 30 min preincubation, Span and Span-Chol (0.1, 1, or 10 μM) caused a concentration-dependent inhibition of SP-stimulated NK_1_R-RLuc8/β-arrestin2-YFP BRET. After a pulsed 4 h preincubation with antagonists, Span had no effect on SP-stimulated NK_1_R-RLuc8/β-arrestin2-YFP BRET at any concentration (Fig. 3, *B* and *C*). In contrast, Span-Chol inhibited SP-stimulated NK_1_R-RLuc8/β-arrestin2-YFP BRET at the two highest concentrations of antagonist (1 and 10 μM).

Similar results were obtained when we measured the effect of Span or Span-Chol on the SP-stimulated change in BRET between NK_1_R-RLuc8 and KRas-Venus (Fig. 3, *D–F*) or Rab5a-Venus (Fig. 3, *G–I*). In control cells, SP caused a decrease in BRET between NK_1_R-RLuc8 and KRas-Venus (Fig. 3, *D–F*), which corresponded to an increase in BRET between NK_1_R-RLuc8 and Rab5a-Venus (Fig. 3, *G–I*). This is consistent with receptor internalization from the plasma membrane (KRas) to early endosomes (Rab5a). After a 30 min preincubation, both Span and Span-Chol inhibited the SP-stimulated change in BRET between NK_1_R-RLuc8 and KRas-Venus (Fig. 3, *D* and *F*) or Rab5a-Venus (Fig. 3, *G* and *I*). However, after a pulsed 4 h preincubation, only Span-Chol inhibited the SP-stimulated change in BRET between NK_1_R-RLuc8 and KRas-Venus (Fig. 3, *E* and *F*) or Rab5a-Venus (Fig. 3, *H* and *I*).

Since alterations in the composition of membrane lipids could artifactually affect BRET between transmembrane and associated proteins, we also studied the effects of a control cholestanol-PEG-biotin probe (Chol). There was no effect of any tested concentration of Chol (0.1, 1, 10 μM) on the SP-induced changes in BRET between NK_1_R-RLuc8 and β-arrestin2-YFP, KRas-Venus, or Rab5a-Venus (Fig. S3).

Our results show that Span-Chol can antagonize the NK_1_R at the plasma membrane to inhibit β-arrestin2 recruitment and receptor endocytosis. This effect is prolonged for up to 4 h, suggesting that some Span-Chol is retained at the plasma membrane despite significant movement of the lipid-anchored antagonist into endosomes (5).

### Lipid-anchored probes traffic from the plasma membrane to endosomal compartments

**Figure 4:**
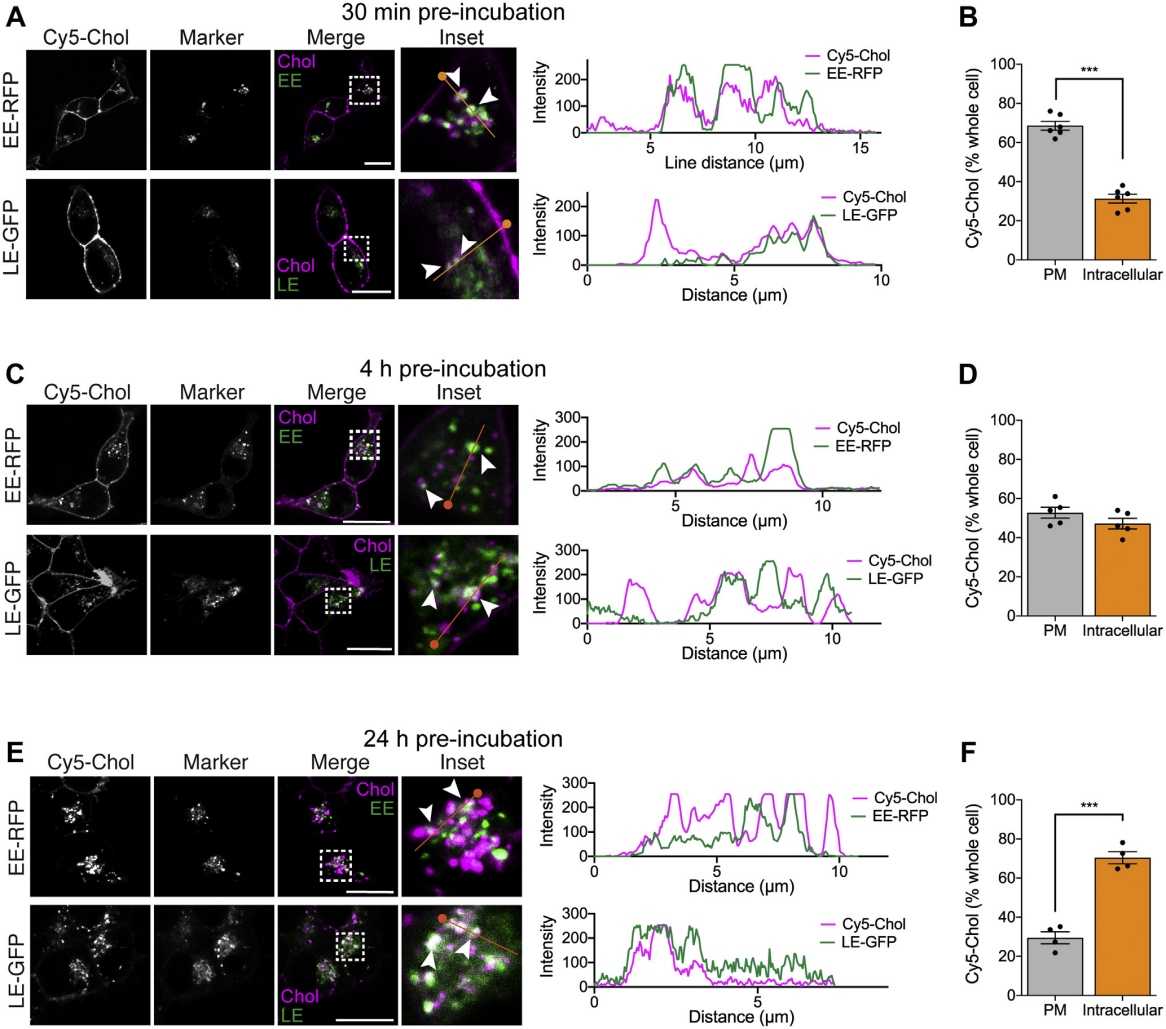
**The cholestanol lipid anchor causes Cy5 movement from the plasma membrane deeper into endosomal pathways over 24 h.** The location of the Cy5-Chol probe (1.5 μM) was determined after 30 min (*A*, *B*), 4 h (*C*, *D*) or 24 h (*E*, *F*) by confocal microscopy in HEK cells labeled with location markers of the endosomal network (CellLight: early endosome(EE)-RFP or late endosome(LE)-GFP) (n=4–6). *A, C, E*, representative, merged and zoomed images of HEK cells with location markers pseudocolored *green*, after 30 min incubation with Cy5-Chol (pseudocolored *magenta*). *Dotted box* indicates zoomed region for inset image. *Arrow heads* indicate coincidence of the Cy5-Chol with the location marker. *Orange line* indicates region highlighted in line scan intensity graph (right panel), with the start of the line indicated by a *circle*. Scale bar, 20 μm. *B, D, F*, the proportion of Cy5-Chol fluorescence at the plasma membrane compared with the rest of the cell (defined as intracellular Cy5). Bars show the grouped mean, and error bars represent S.E.M. of grouped cells from 4 to 6 independent experiments. ∗∗∗*p* < 0.001, unpaired *t*-test.

We have previously demonstrated that the Cy5-Chol probe accumulates in early endosomes in HEK293 cells after 4 h, as indicated by colocalization with Rab5a (5). However, we still observe effects of Span-Chol at the plasma membrane at this time point, and the distribution of lipid-anchored probes into other endosomal signaling compartments (*i.e.*, late endosomes) has not been investigated. We therefore set out to map the location of Cy5-Chol over short and longer timescales. Previous studies have shown accumulation of a fluorescent analog of Span-Chol (Cy5-Span-Chol) in NK_1_R-positive endosomes in cells stimulated with SP (5). Here, we aimed to further define the role of the lipid anchor in influencing the cellular distribution of cargo when not engaged with its receptor target (Fig. 4).

HEK293 cells were infected with CellLight fluorescent fusion proteins resident to endocytic compartments, including early endosomes (EE-RFP) and late endosomes (LE-GFP). The distribution of Cy5-Chol (1.5 μM) was examined in live cells by confocal microscopy due to a loss of probe fluorescence that occurs when using standard fixation approaches. Using this approach, some of the finer endosomal structures are less evident in comparison with antibody staining of Rab GTPases in fixed cells (26). Nevertheless, live cell imaging still provides valuable insights into the distribution of lipidated probes within the endosomal network over time. After a 30 min preincubation, Cy5-Chol fluorescence was readily observed at the plasma membrane, in early endosomes (EE-RFP) and late endosomes (LE-GFP) (Fig. 4*A*). A much higher proportion of Cy5-Chol was observed at the plasma membrane, compared with intracellular compartments (Fig. 4*B*). We then assessed distribution of Cy5-Chol after a pulsed incubation protocol (30 min incubation with Cy5-Chol, wash, 3.5 h recovery; 4 h total). We observed coincident detection of Cy5-Chol with markers of early endosomes (EE-RFP) and late endosomes (LE-GFP) (Fig. 4*C*). This correlated with a change in the overall distribution of Cy5-Chol in the cell, with similar fluorescence observed at the plasma membrane and within intracellular compartments (Fig. 4*D*). To determine the long-term intracellular distribution of a lipidated probe, HEK293 cells were incubated with Cy5-Chol for 24 h (Fig. 4, *E* and *F*). After 24 h we still detected Cy5-Chol codistribution with reporters for early endosomes (EE-RFP) and late endosomes (LE-GFP) (Fig. 4*E*). However, the relative distribution of Cy5-Chol over the whole cell was enriched in intracellular compartments compared with the plasma membrane (Fig. 4*F*).

Together, these data indicate that the internalized cholestanol-conjugated reporter resides within the endocytic pathway for sustained periods. Over time, the amount of Cy5-Chol at the plasma membrane decreases, which corresponds with a movement of Cy5-Chol further into the endosomal network. These findings support the use of sterol-based lipid anchors for targeting ligands to populations of endosomal GPCRs.

### Only free antagonist completely blocked plasma membrane NK_1_R signaling

We then investigated in detail the capacity of Span versus Span-Chol to target NK_1_R signaling in different cellular regions. Our previous analysis had focused only on ERK activity, showing selective inhibition of nuclear ERK by Span-Chol (versus Span) (5). This is because only endosomal NK_1_R can increase nuclear ERK in response to SP (5). Here, we used an expanded toolbox of targeted FRET biosensors to follow the signaling of the NK_1_R in live cells as the receptor moves from the plasma membrane to early endosomes.

SP stimulation of NK_1_R at the cell surface causes activation of Gα_q_ signaling, which is limited to the plasma membrane (5, 14). NK_1_R-Gα_q_ stimulates phospholipase C (PLC)-dependent formation of inositol trisphosphate (InsP_3_) and diacylglycerol (DAG). InsP_3_ causes the transient release of Ca^2+^ (Fig. 2), and then both DAG and Ca^2+^ activate protein kinase C (PKC). PKC can then activate adenylyl cyclase (AC) to increase cAMP (Fig. S1).

**Figure 5:**
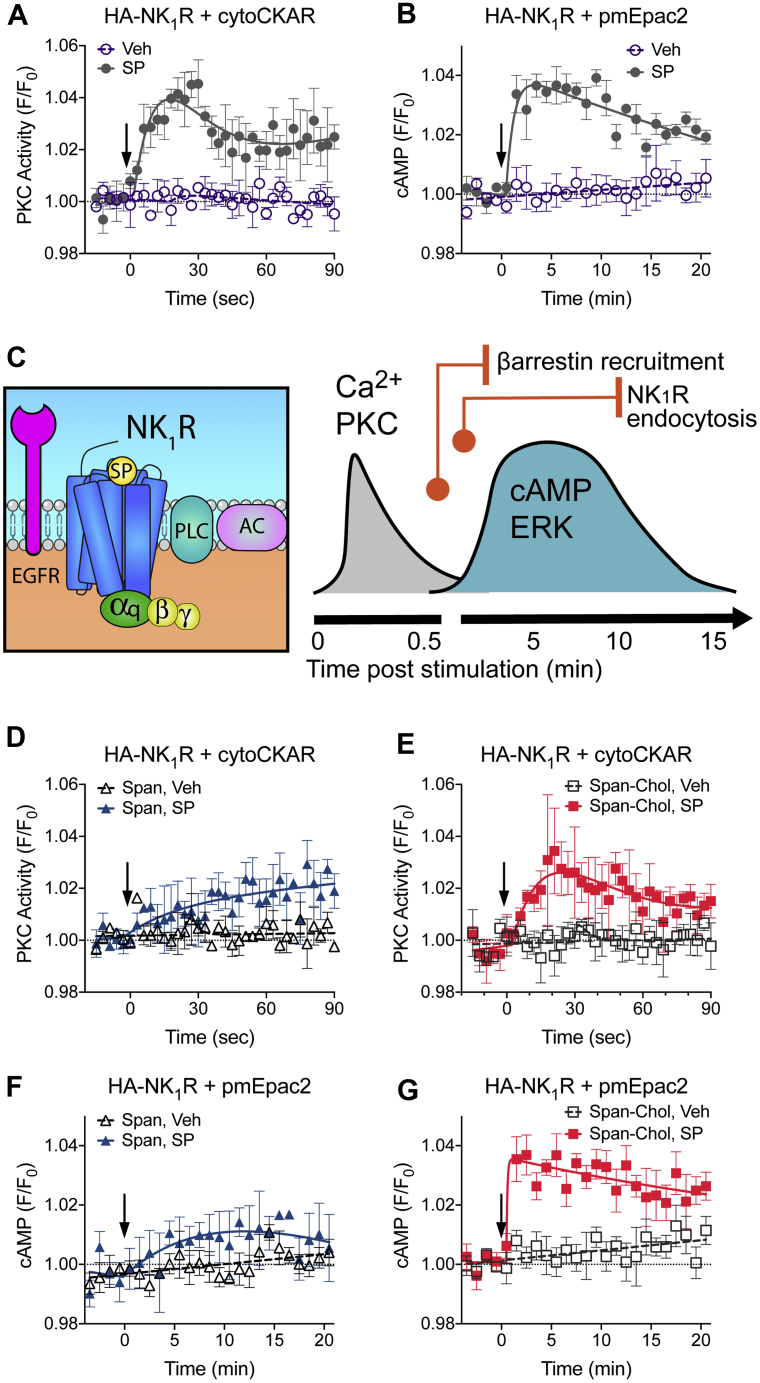
**Only Span inhibits all NK**_**1**_**R signaling from the plasma membrane.** After a 4 h preincubation Span, but not Span-Chol, blocks SP-stimulated transient increases in PKC activity and cAMP (n = 3). *A* and *B*, HEK cells transfected with HA-NK_1_R and cytoCKAR (*A*) or pmEpac2 (*B*) were stimulated with vehicle (0.0001% v/v MilliQ H_2_O) or 1 nM SP and signaling was measured over time. *C*, Cartoon of the sequence of events following NK_1_R stimulation at the plasma membrane. *Orange circles* indicate the time at which a regulatory event starts, and vertical *orange lines* indicate when it reaches a plateau. Signaling is represented by *black lines*. *D–G*, HEK cells transfected with HA-NK_1_R and cytoCKAR (*D–E*) or pmEpac2 (*F–G*) were pretreated with 1 μM Span (*D*, *F*) or Span-Chol (*E*, *G*) for 4 h prior to addition of vehicle (0.0001% v/v MilliQ H_2_O) or 1 nM SP. Data are expressed as the FRET relative to the baseline FRET (F/F_0_). *Arrows* indicate time of vehicle/SP addition. Symbols show the mean, and error bars S.E.M. of grouped cells from three independent experiments.

We can measure changes in these transient signals from the NK_1_R at the plasma membrane of live cells. In HEK293 cells transfected with HA-NK_1_R and a PKC FRET biosensor (cytoCKAR), fast imaging shows a transient increase in PKC activity in response to SP, which declined to a steady-state level by 30 s following receptor stimulation (Fig. 5*A*). In HEK293 cells transfected with HA-NK_1_R and a plasma membrane cAMP FRET biosensor (pmEpac2), this transient PKC signal was followed by a slightly delayed but also transient increase in cAMP at the plasma membrane in response to SP. With a peak at ∼5 min, the cAMP response then declined slowly toward baseline (Fig. 5*B*). This high-resolution examination of localized signaling allowed us to assemble a timescale of events at the plasma membrane following NK_1_R stimulation (Fig. 5*C*). The activated receptor causes a fast peak of both Ca^2+^ and PKC activity in the first 30 s, which overlaps the start of the transient cAMP and cytosolic ERK signals. The peak of cAMP and cytosolic ERK signaling coincides with a plateau in the recruitment of β-arrestins (2–5 min post receptor stimulation) (5). The cAMP and cytosolic ERK signals then decline back toward baseline, which coincides with a plateau in the internalization of NK_1_R to early endosomes (10–15 min post receptor activation) (5).

Using this timescale of events at the cell surface, we assessed the relative impact of Span versus Span-Chol on NK_1_R signaling from the plasma membrane. We used a continuous incubation protocol for 4 h, so as not to wash away the free Span. This allows us to compare the spatial efficacy of both antagonists. A 4 h preincubation of the cells with Span inhibited the fast peak of PKC activity in response to SP, but there was no effect of Span-Chol on this signal (Figs. 5, *D* and *E*, S4, *A* and *B*). Similarly, a 4 h preincubation of the cells with Span inhibited the SP-induced increase in cAMP at the plasma membrane, with no effect of preincubation with Span-Chol on this signal (Figs. 5, *F* and *G*, S4, *C* and *D*).

These data suggest that while Span-Chol effectively blocks Ca^2+^ mobilization (Fig. 2), it is unable to block the PKC and cAMP signals activated by the SP-stimulated NK_1_R at the plasma membrane. In contrast, free Span inhibits all signaling of the plasma-membrane-localized receptor (Ca^2+^, PKC and cAMP).

### Only a lipid-anchored antagonist can inhibit endosomal NK_1_R signaling

Following NK_1_R activation by SP, there is a rapid recruitment of β-arrestins and internalization of the receptor to early endosomes. Here, the NK_1_R also colocalizes with Gα_q_ and causes a sustained increase in PKC, cAMP, and ERK (5) (Fig. S1).

**Figure 6:**
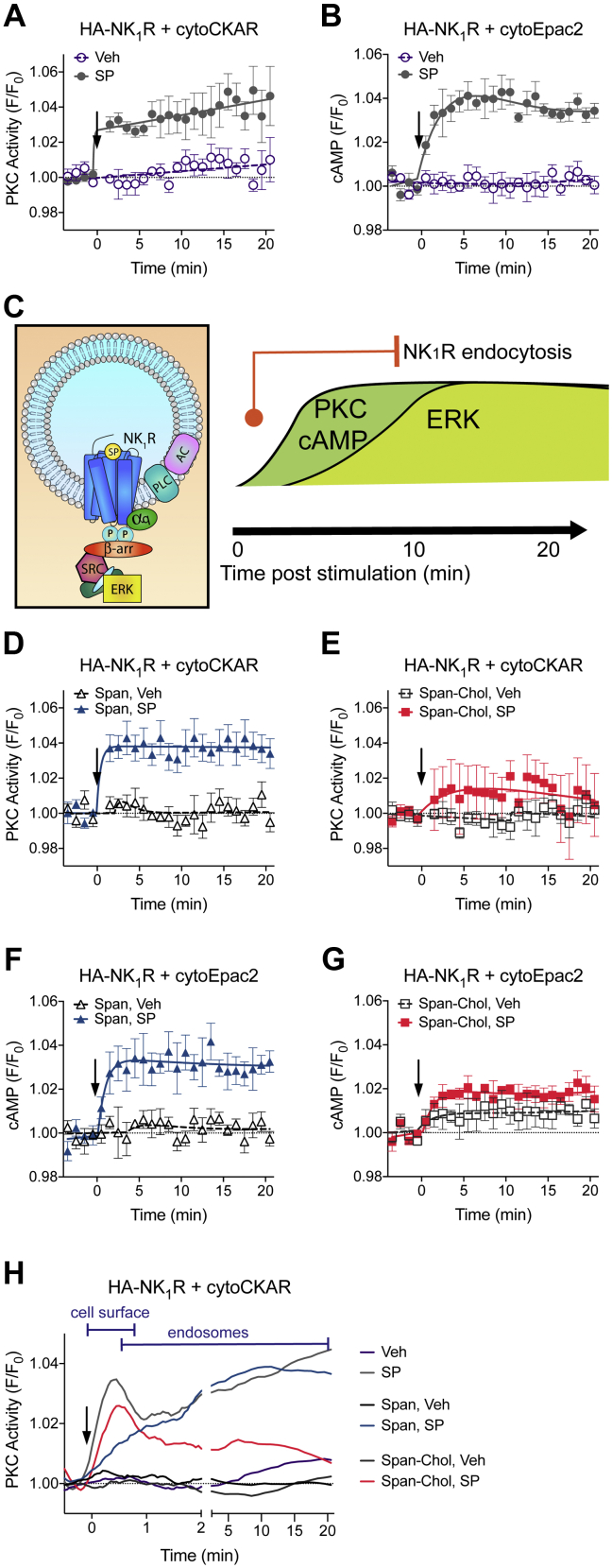
**Only Span-Chol, and not Span, inhibits NK**_**1**_**R signaling from endosomes.** After a 4 h preincubation Span-Chol, but not Span, blocks SP-stimulated sustained increases in PKC and cAMP (n = 3). *A* and *B*, HEK cells transfected with HA-NK_1_R and cytoCKAR (*A*) or cytoEpac2 (*B*) were stimulated with vehicle (0.0001% v/v MilliQ H2O) or 1 nM SP and signaling was measured over 20 min. *C*, cartoon of the sequence of events in endosomes following NK_1_R stimulation. β-arr, β-arrestin. The *orange circle* indicates the time at which internalization starts, and the *vertical orange line* indicates when it reaches a plateau. Signaling is represented by black lines. *D–H*, HEK cells transfected with HA-NK_1_R and cytoCKAR (*D–E*) or cytoEpac2 (*F–G*) were pretreated with 1 μM Span (*D, F*) or Span-Chol (*E, G*) for 4 h prior to addition of vehicle (0.0001% v/v MilliQ H2O) or 1 nM SP. Symbols show the mean, and error bars represent S.E.M. of grouped cells from three independent experiments. *H*, smoothed time course traces showing the change in effectiveness of Span versus Span-Chol at blocking SP-stimulated PKC signaling as the NK_1_R transitions from the plasma membrane (data taken from Fig. 5*A*) to endosomes (data from Fig. 6*A*). Data are expressed as the FRET relative to the baseline FRET (F/F_0_). *Arrows* indicate time of vehicle/SP addition.

We can measure changes in these sustained signals from the NK_1_R in endosomes of live cells. In HEK293 cells transfected with the HA-NK_1_R and cytoCKAR, high content imaging over 20 min showed a steep increase in PKC activity by 1 min, which was sustained over the measurement period (Fig. 6*A*). Similarly, in HEK293 cells transfected with HA-NK_1_R and a cytosolic cAMP FRET biosensor (cytoEpac2), we observed a prolonged increase in cAMP, which peaked by 2 min and was sustained over the 20 min period (Fig. 6*B*). This high-resolution examination of localized signaling allowed us to define a timescale of events at early endosomes following NK_1_R stimulation (Fig. 6*C*). The activated NK_1_R rapidly traffics to early endosomes that also contain Gα_q_ within 1 min post receptor stimulation (5). This movement corresponds with a rapid and sustained increase in PKC activity and cAMP over very similar timescales. The sustained increase in nuclear ERK mediated by endosomal NK_1_R is slightly delayed and peaks ∼10 min post receptor stimulation (5).

To compare the effect of Span versus Span-Chol on NK_1_R signaling from early endosomes, we used a 4 h continuous incubation protocol. Under these conditions, Span had no effect on the SP-induced increase in PKC or cAMP (Figs. 6, *D* and *E*, S4, E–H). In contrast, preincubation for 4 h with Span-Chol inhibited SP-induced PKC and cAMP signaling (Figs. 6, *F* and *G*, S4, E–H).

Given the clear time distinction between the two PKC events stimulated by the plasma membrane versus endosomal NK_1_R, we can visualize the changing spatial efficacies of the two antagonists (Figs. 6*H*, S4*I*). Under control conditions, spatiotemporal coordination of PKC activity is observed, where SP causes an initial peak in PKC activity from the plasma membrane NK_1_R and then a steady increase in PKC activity from endosomal NK_1_R. Signaling waves of this nature may be mediated due to spatially dependent activation of differentially localized PKC isoforms (27, 28).

A long preincubation with Span inhibits the PKC signal from cell surface NK_1_R but has no effect on the PKC signals activated by endosomal NK_1_R. In contrast, long preincubation with Span-Chol has no effect on the initial PKC signal from the activated cell surface NK_1_R, but selectively inhibits signals from the endosomal NK_1_R. These data suggest that free Span effectively inhibits plasma-membrane-delimited signaling but is unable to block signaling driven by intracellular NK_1_R. In contrast, Span-Chol favors inhibition of PKC and cAMP signals activated by SP-NK_1_R from endosomes.

### The three-pronged mechanism of action of Span-Chol contributes to its long-lasting antinociceptive actions

We have previously demonstrated that blockade of endosomal (compared with plasma membrane) NK_1_R causes much more effective antinociception (5, 17). In preclinical models of pain, the analgesic effect of Span-Chol was maintained for up to 6 h (5). However, it is unknown for how long this analgesic effect is sustained. We recently examined the analgesic effect of an NK_1_R antagonist (aprepitant) over a 24 h period after directly delivering it to endosomes and found that antinociception was maintained for 6 h, before dropping back to baseline (17).

**Figure 7:**
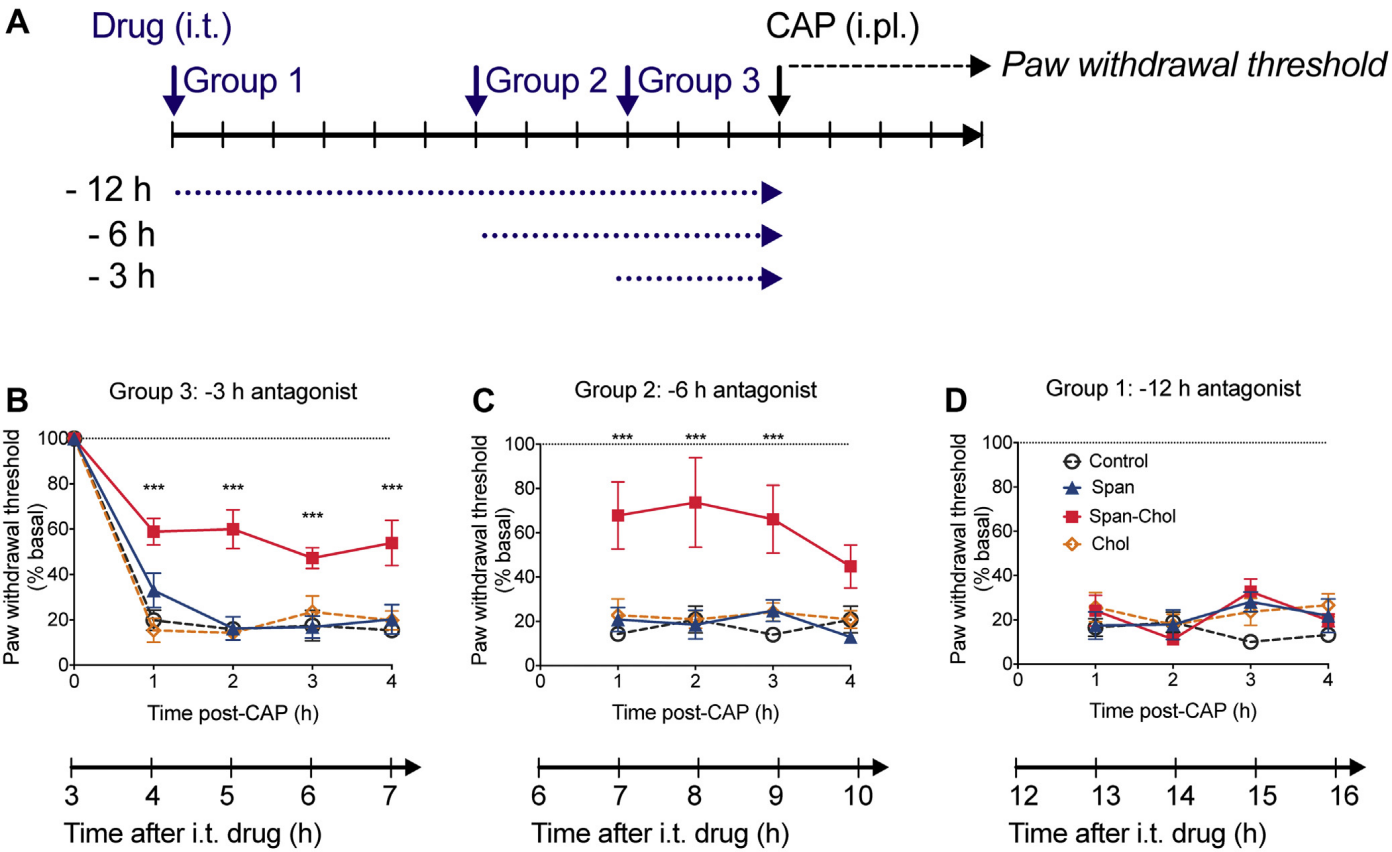
**Span-Chol causes a prolonged antinociception in mice up to 9 h post administration.** The analgesic effects of Span-Chol were assessed over 16 h in a mouse model of mechanical nociception (n = 6). *A*, illustration of the experimental protocol: Span (50 μM), Span-Chol (50 μM), Chol (50 μM), or vehicle (1% v/v DMSO/saline) was administered by intrathecal (i.t.) injection to three different groups of mice. The mice were left for 12 h (group 1), 6 h (group 2), or 3 h (group 3) before intraplantar (i.pl.) injection of capsaicin (CAP, 5 μg, 10 μl). Paw withdrawal responses to stimulation with von Frey filaments were measured hourly for 4 h. *B–D*, Paw withdrawal responses measured in the different groups of mice at 3 h (*B*), 6 h (*C*), or 12 h (*D*) after i.t. drug administration. Data are expressed relative to the baseline paw withdrawal threshold established for each mouse at the start of the experiment. Symbols show the mean, and error bars represent S.E.M. from 6 mice. ∗∗∗*p* < 0.001 compared with mice that received i.t. vehicle, two-way ANOVA with Bonferroni posttest.

The three-pronged mechanism of Span-Chol identified in this study (higher local concentration at membranes, decreased receptor internalization, and complete inhibition of endosomal signaling) suggested that Span-Chol could provide prolonged pain relief. To evaluate this possibility, Span, Span-Chol, or controls were administered by intrathecal injection to three different groups of mice (Fig. 7*A*). Each group received an injection of capsaicin into the plantar surface of the left hindpaw at different times after intrathecal administration of the antagonists (*i.e.*, capsaicin injected 3 h, 6 h, or 12 h after antagonist administration). Mechanical nociception was evaluated by measurement of paw withdrawal responses to stimulation of the plantar surface with calibrated von Frey filaments every hour for 4 h after administration of capsaicin. As mechanical nociception to capsaicin was measured over exactly the same time period for all groups (4 h), this allowed us to build a timescale of the analgesic effect of Span-Chol over a cumulative 16 h period (Fig. 7).

In control mice receiving intrathecal vehicle, capsaicin caused a prolonged allodynia over 4 h (Fig. 7, *B–D*). Neither free Span nor the Chol control had any effect at any time tested. In contrast, Span-Chol had a marked antinociceptive action that was already present at 4 h post intrathecal injection and was fully maintained for 9 h after intrathecal injection. Thus, intrathecal delivery of Span-Chol resulted in a long duration of antinociception.

## Discussion

The NK_1_R is expressed throughout the nervous, immune, digestive, respiratory, and urogenital systems, where it regulates pain, inflammation, motility, and secretion (29, 30, 31, 32, 33, 34). In the context of pain, noxious stimuli evoke the release of SP from peripheral and central projections of primary afferent neurons. In the dorsal horn of the spinal cord, SP then activates the NK_1_R on second-order spinal neurons to mediate pain transmission (29). Despite this clear role in pain transmission, there has been limited clinical success for drug discovery programs targeting the NK_1_R for chronic pain (29, 35). Previously, we reported that pain transmission is dependent on sustained signaling from the NK_1_R internalized to endosomes and that we could improve analgesic effect and duration in preclinical models of pain by specifically blocking endosomal (and not cell surface) NK_1_R (5, 17). Here, in addition to blockade of endosomal NK_1_R, we have identified two further effects of a lipid-anchored NK_1_R antagonist that contribute to its increased efficacy. First, we find that the addition of a lipid anchor causes a fourfold increase in the local concentration of a probe directly above the cell membrane. Second, although the probe quickly internalizes, 29.6% of the lipid-anchored probe remains at the plasma membrane even 24 h after administration. This residual plasma membrane localization facilitates an inhibition of NK_1_R trafficking to endosomes. Together, this three-pronged mechanism—increased local concentration, inhibition of NK_1_R trafficking to endosomes, and sustained blockade of endosomal signaling—contributes to the prolonged analgesic effects of lipidated antagonists in preclinical models of pain.

Cholestanol has a high affinity for sterol-rich microdomains of the outer leaflet of lipid bilayers. The binding of cholestanol to the sterol-rich microdomains then promotes internalization into endosomal compartments (36). Cholestanol has also been used to target an inhibitor of the recycling endopeptidase, β-site amyloid precursor protein cleaving enzyme 1 (BACE-1), to early endosomes (37). A lipid conjugated, but not a free antagonist, inhibited the cleavage of amyloid precursor protein at the BACE-1 ectodomain, a rate-limiting step in the production of the β-amyloid peptide. We subsequently used this approach to target an NK_1_R antagonist to endosomes and observed a prolonged and seemingly selective inhibition of endosomal compared with plasma membrane NK_1_R signaling (5). However, with further investigation we now find that the effects of a cholestanol-conjugated NK_1_R antagonist are not limited to blockade of endosomal signaling. In addition to delivery to endosomes, we found that cholestanol conjugation causes a prolonged increase in the partitioning of the cargo into the plasma membrane. This is consistent with previous studies using Cy5-Chol or Cy5-Span-Chol that show fluorescent probe distribution in both endosomes and the plasma membrane over time (5). Increasing the lipophilic properties of soluble drugs, such as GPCR antagonists, can increase their association with membranes and may therefore enhance their local potency (24, 38). Here we found that the addition of cholestanol caused approximately fourfold increase in the concentration of Cy5 directly above the plasma membrane when we measured Cy5-Chol compared with Cy5-OEt concentrations using FCS. Although we did not directly measure the concentration of Span-Chol itself, a previous study reported a twofold local enrichment of the concentration of a GPCR ligand at the surface of cells transfected with the target GPCR, compared with nontransfected cells (22). This increase in local concentration was achieved without any change in the lipophilic properties of the ligand itself. As such, we would expect the local concentration of a lipid-anchored GPCR ligand to be *at least* fourfold higher at the surface of target cells. Consistent with this, we observed a corresponding increase in the potency and affinity of Span-Chol as compared with Span. This suggests that the blockade of endosomal signaling of the NK_1_R by Span-Chol is not only due to its spatial distribution but could also be influenced by a high local concentration of Span-Chol at endosomal membranes.

Given the inherent ability of cholestanol conjugation to cause a prolonged increase in partitioning into membranes, it is important to map where the probes travel in cells. After initial incorporation into the plasma membrane, cholestanol probes translocate from the plasma membrane to endosomes. Within endosomes, cholestanol-conjugated antagonists inhibit the endosomal signaling of the NK_1_R that underlies persistent excitation of spinal neurons and pain transmission (5). Consistent with our previous study, we find that Cy5-Chol is rapidly internalized into the endosomal network where it is codistributed with early and late endosomes. Despite this large movement of the Cy5-Chol into the cell, some of the probe remains at the plasma membrane even after 24 h. This persistent partitioning of Cy5-Chol into the plasma membrane led us to look for an effect of Span-Chol on NK_1_R endocytosis. BRET receptor trafficking studies revealed that up to 4 h after a pulse administration of Span-Chol, the lipid-anchored antagonist could inhibit receptor trafficking by blocking the recruitment of β-arrestins and therefore, subsequent receptor internalization to early endosomes. This inhibition of receptor movement into endosomes likely contributes to the overall decrease in endosomal signaling.

Due to the effective blockade of plasma membrane NK_1_R calcium signaling (Fig. 2), β-arrestin recruitment, and receptor internalization (Fig. 3) by Span-Chol, we then examined the ability of the lipidated antagonist to affect other plasma-membrane-dependent NK_1_R signaling pathways. Using an expanded toolbox of targeted FRET biosensors, we have mapped the signaling of the NK_1_R as it traffics from the plasma membrane to endosomes. By comparing cAMP production detected by cytosolic and plasma-membrane-localized cAMP FRET biosensors, and delineating the temporal profiles of cytosolic PKC activity (acute versus sustained phases), we can show that Span-Chol is more effective at inhibiting endosomal-selective NK_1_R signaling over sustained time periods. After 4 h of continuous administration, we find that Span effectively blocks all signaling from the plasma-membrane-localized NK_1_R, but has no effect on signaling from the endosomal NK_1_R. In contrast, Span-Chol was unable to block PKC or cAMP signals from the plasma membrane NK_1_R but blocked all signaling from the endosomal NK_1_R. It is interesting that Span-Chol appears to block NK_1_R Ca^2+^ signaling and receptor internalization, but not cAMP or PKC signaling from the plasma membrane. GPCRs are highly flexible proteins that fluctuate between many different conformational states (39, 40). They may adopt different conformations at the plasma membrane versus in endosomes due to large differences in the curvature of the two membranes, the composition of the associated membrane lipids, and allosteric effects of associations with receptor signaling complexes (40, 41, 42, 43, 44, 45, 46). This could effectively facilitate slightly different binding orientations for Span-Chol in the two locations and perhaps allow location-biased antagonism. Alternatively, the enclosed and small volume of an endosome could effectively result in a much higher local concentration of the antagonist compared with the open and large volume of the extracellular space. The end result is that at the plasma membrane, Span-Chol is apparently more effective at inhibiting receptor internalization and Ca^2+^ signaling than inhibiting cAMP and PKC signaling. Signaling, in contrast to receptor internalization, is typically a highly amplified event. The recruitment of β-arrestins to a receptor, and subsequent β-arrestin-mediated internalization, is generally considered a low amplification event (47). In contrast, a single GPCR can activate multiple G proteins, which in turn switch on (or off) kinases or enzymes. For example, it is estimated that a single photon of light hitting a photosenstitive GPCR can activate between 16 and 60 G proteins, which in turn activate phosphodiesterases to hydrolyze 2000–72,000 molecules of cGMP (48). Small differences in the local concentration of Span-Chol or even its binding orientation could therefore have dramatic effects on receptor trafficking without seeming to affect downstream signaling (such as cAMP, PKC, ERK) from the receptor at the plasma membrane. Further studies involving, for example, direct measurement of NK_1_R-G protein coupling at the plasma membrane compared with endosomes, may provide further insight into the mechanism of action for Span-Chol relative to Span.

We previously showed that Span-Chol could inhibit sustained pain transmission for up to 6 h following administration in preclinical models (5). Here we extended this analysis to show that the analgesic effects of Span-Chol are retained for >9 h following administration. This study demonstrates that lipidation is a viable approach, not only for enhancing membrane affinity of soluble GPCR antagonists, but also for targeting NK_1_R signaling pathways of pathophysiological importance. Furthermore, this novel approach improves the pharmacological properties of an otherwise less potent NK_1_R antagonist and results in potent and selective inhibition of signaling events associated with central pain transmission.

One explanation for the failure of previous drug discovery programs targeting the NK_1_R for chronic pain is that they have only targeted plasma-membrane-localized NK_1_R. Until very recently, GPCRs were only considered to be active at the cell surface, and therefore most drugs targeting GPCRs are not required to cross the plasma membrane. There is now clear evidence to show that activation of receptors in endosomes (compared with the cell surface) encodes for distinct physiological outcomes (5, 8, 49, 50, 51, 52). It is therefore important to consider the subcellular location of a target GPCR, and whether they reside in, or are delivered to, a particular location. For example, the β_1_-adrenoceptor is localized to two distinct pools in cells: one at the cell surface and a second at the Golgi (16). Golgi-localized signaling of the β_1_-adrenoceptor requires a preexisting pool of receptors (*i.e.*, they are not delivered to the Golgi following internalization from the cell surface). In this case, as these two receptor populations are distinct, a targeting strategy involving direct delivery would be better suited than one that also facilitates inhibition of cell surface receptor endocytosis. In contrast, the two NK_1_R receptor pools at the cell surface and endosomes are linked by receptor internalization. As such, blockade of the endosomal pool is further enhanced by preventing movement of the pool at the cell surface into the endosomal network. This could explain the prolonged analgesic activity of a lipidated-NK_1_R antagonists versus an antagonist directly delivered to endosomes (5, 17) (Fig. 7).

Whether preventing NK_1_R internalization (in addition to inhibition of endosomal NK_1_R signaling) would be of benefit in situations of chronic pain is uncertain. In patients suffering from chronic visceral pain, the NK_1_R is no longer available at the cell surface, but is instead found principally within intracellular compartments (35). In this case, as for the Golgi-localized β_1_-adrenoceptor, there may be no added benefit of blocking receptor internalization from the cell surface. Future studies will need to directly compare different methods of endosomal drug delivery and their resulting efficacy in a variety of disease models. Identifying additional mechanisms that contribute to the increased preclinical efficacy of lipid-anchored NK_1_R antagonists is an important step toward understanding how we can effectively target intracellular GPCRs in disease.

## Experimental procedures

### Probes

The tripartite probes Span-Chol, Cy5-Chol, Cy5-OEt, and Chol were synthesized as described previously (5, 8). Tetramethylrhodamine (TAMRA)-labeled SP (SP-TAMRA) was synthesized by GL Biochem (Shanghai, China).

### cDNAs

Rat NK_1_R-GFP, HA-NK_1_R, and NK_1_R-RLuc8 have been described (14, 53). SNAP-NK_1_R was from Cisbio. CytoCKAR (Addgene plasmid 14,870) was from A. Newton (54). CytoEpac2-camps was from M. Lohse (University of Wurzburg, Germany) (9), and pmEpac2-camps was from D. Cooper (University of Cambridge, UK) (55). KRas-Venus (56) and Rab5a-Venus (57) were from N. Lambert. β-arrestin 2-YFP was from M. Caron (University of North Carolina).

### Cell culture and transfection

HEK293 cells (ATCC, negative for *mycoplasma* contamination) were maintained in Dulbecco’s modified Eagle’s medium (DMEM) supplemented with 5% (v/v) FBS. HEK293 cells were transfected using linear polyethyleneimine. HEK293-FlpIn cells stably expressing rat HA-NK_1_R (HEK-NK_1_R) and SNAP-NK_1_R (HEK-SNAP-NK_1_R) have been described (5, 15). HEK-NK_1_R and HEK-SNAP-NK_1_R cells were maintained in DMEM supplemented with 10% (v/v) FBS and 100 μg/ml Hygromycin B. All assay dishes and plates were coated with poly-D-lysine (5 μg/cm^2^).

### Fluorescence correlation spectroscopy (FCS)

FCS measurements were made using a Zeiss LSM510Meta ConfoCor 3 microscope fitted with a c-Apochromat 40x NA 1.2 water immersion objective lens (58). Cy5 was excited using a 633 nm HeNe laser, with emission collected through a 650LP filter and the pinhole diameter of 1 Airy unit. Prior to each experiment, Cy5 NHS ester (GE healthcare, Buckingham, UK) was used to calibrate the 633 nm detection volume using a literature value for diffusion coefficient (D) of 3.16 x 10^-10^ m^2^/s, as described (22, 58).

HEK-NK_1_R-SNAP was plated on Nunc Lab-Tek 8-well coverglasses (SLS, Nottingham, UK). After 24 h, Cy5-Chol and Cy5-OEt were prepared in HBSS and cells were incubated with a 10 nM solution of each ligand for 10 min at 37ºC in a final volume of 400 μl. A reference confocal image of each cell was captured, before positioning the FCS detection volume in x-y using a live confocal image. A fluorescence intensity scan in the z direction was used to determine the position of the plasma membrane, and the focal point was positioned at defined distances above this point using the microscope’s harmonic z-drive. FCS fluctuations were recorded at each point (ex λ: 633 nm HeNe, em λ: LP650 nm filter) for 20 s, at a laser power of ∼1 kW/cm^2^.

Probe dwell times and particle numbers were obtained from subsequent autocorrelation analysis of the fluctuations, performed with a 1 component, 3D Brownian model fit incorporating a triplet state pre-exponential using Zeiss 2010 Black software (22). Probe concentration and diffusion coefficients were calculated from measurements of dwell time and particle number, respectively, using the dimensions of the detection volume calculated from the Cy5 calibration data.

### High-content fluorescent competition binding

HEK-NK_1_R cells in black optically-clear 96 well plates were grown to 80% confluency. Cells were pretreated at 37°C with increasing concentrations of Span or Span-Chol for the indicated times, followed by an EC_50_ concentration (0.5 nM) of SP-TAMRA. Total binding was determined by preincubation with a vehicle control (0.1% v/v DMSO). Cell nuclei were stained with Hoechst 33,342 (1 μg/ml, 30 min, 37°C). Images were acquired using an ImageXpress Ultra confocal high-content plate reader (Molecular Devices, Sunnyvale, CA, USA) with Fluor 40x NA0.6 objective and the pinhole set to 4. Cells were imaged using the 405 nm and 561 nm laser excitations for Hoechst (DAPI filter) and TAMRA (Texas Red filter), respectively. The experiment was performed in triplicate with four fields of view imaged per well. Images were analyzed with MetaXpress 2.0 software (Molecular Devices), using an automated granularity module with the granule range set to 5–10 μm and intensity thresholds for granule classification set for each experiment based on the positive and negative controls (*i.e.*, total and nonspecific binding). A nuclear count from the Hoechst 33,342 image was obtained and the granularity module calculated the average intensity per cell, as previously described (23, 59). Data were fit with a competitive binding, one site, fit logIC_50_ model.

### Confocal imaging

To identify endosomal compartments, HEK293 cells were transduced with fluorescent fusion proteins using CellLight BacMam 2.0 virus (Life Technologies) for 16 h. CellLight fusion proteins used were as follows: early endosome-RFP, late endosome-GFP. Cells were equilibrated in Hanks’ Balanced Salt Solution (HBSS) for 30 min prior to imaging.

Images were obtained using a Leica TCS SP8 Laser-scanning confocal microscope with HCX PL APO 40x (NA 1.30) and HCX PL APO 63x (NA 1.40) oil objectives in a humidified and temperature-controlled chamber at 37°C. For each cell, three baseline images were captured (4–6 optical sections) before addition of Cy5-Chol (1.5 μM). Cells were imaged at different time points following probe addition, as indicated.

Imaging was performed on at least three different days with separate drug preparations. Line scan intensity was processed using the FIJI distribution of Image J (60). The proportion of Cy5 fluorescence at the plasma membrane compared with the rest of the cell was calculated as a percentage of the raw integrated density of the total cell area.

### Measurement of intracellular Ca^2+^

HEK-NK_1_R cells in 96-well plates were washed with calcium buffer (10 mM HEPES, 0.5% w/v BSA, 10 mM D-glucose, 2.2 mM CaCl_2_ 1.18 mM MgCl_2_, 2.6 mM KCl, 150 mM NaCl, 4 mM probenecid, 0.05% v/v pluronic acid F127; pH 7.4) and then loaded with 1 μM Fura-2 AM ester (Life Technologies) in calcium buffer for 45 min at 37°C. For short preincubation with the antagonist, increasing concentrations of Span or Span-Chol were incubated with the cells for 30 min during Fura-2 AM loading. For longer preincubation with the antagonist, cells were incubated with increasing concentrations of Span or Span-Chol for the indicated time periods prior to Fura-2 AM loading.

Calcium was measured using a FlexStation 3 plate reader (Molecular Devices). Fluorescence (excitation: 340 nm and 380 nm; emission: 520 nm) was measured at 4 s intervals for a total of 45 s. After establishing baseline fluorescence, cells were stimulated with vehicle, 1 nM SP, or 1 μM ionomycin (to obtain a maximal response). SoftMax Pro (v5.4.4) software was used to calculate the area under the curve from the kinetic data from at least four experiments performed in duplicate.

### Receptor trafficking using BRET

HEK293 cells in 10 cm dishes were cotransfected with 1 μg of NK_1_R-RLuc8 and 4 μg β-arrestin 2-YFP, KRas-Venus or Rab5a-Venus. After 24 h, cells were replated in 96-well white opaque culture plates (CulturPlate-96; PerkinElmer). Forty-eight h after transfection, cells were pretreated with antagonists. For short preincubations, cells were incubated with increasing concentrations of Span, Span-Chol, or Chol in HBSS for 30 min. For “pulsed” long preincubations, cells were incubated with increasing concentrations of Span, Span-Chol, or Chol for 30 min, washed, media was replaced for 3 h, prior to equilibration for 30 min in HBSS (4 h total). Coelenterazine h (Promega) was added at a final concentration of 5 μM, and the cells were incubated for a further 5 min.

The BRET baseline was measured every 1 min for 4 min, before addition of vehicle or 1 nM SP, with BRET measurements continued every 1 min for 25 min. BRET was measured using a PHERAstar Omega microplate reader (BMG Labtech) with sequential integration of the signals detected at 475 ± 30 nm and 535 ± 30 nm with filters with the appropriate band pass. Data are shown as the BRET ratio (calculated as the ratio of the YFP/Venus signal to the RLuc8 signal) expressed as the SP-induced change in BRET (corrected for vehicle) for time course graphs. Curve fitting of time course data used exponential equations in GraphPad Prism version 8.4.3 (plateau followed by one-phase association for Rab5a and β-arrestin2 BRET or one-phase decay for KRas BRET). The plateau was derived from the curve fit for each independent experiment and is shown relative to the control SP response (BRET/BRET_SP_) for bar graphs. Normal distribution of the data was confirmed using normality (QQ) plots in GraphPad Prism prior to statistical analysis.

### Spatial PKC and cAMP using high-content and confocal ratiometric FRET imaging

High-content ratiometric FRET imaging was performed as described previously (61). HEK293 cells in black, optically clear 96-well plates were grown to 70% confluency before cotransfection with 55 ng/well HA-NK_1_R and 40 ng/well cytoCKAR, pmEpac2, or cytoEpac2 for 48 h. Before the experiment, cells were partially serum-restricted overnight in 0.5% (v/v) FBS DMEM. On the day of the experiment, cells were preincubated with Span or Span-Chol (both 1 μM) for 4 h before the medium was replaced with HBSS and cells were equilibrated for 30 min at 37°C. High-content fluorescence imaging was performed using the INCell 2000 Analyzer with a Nikon Plan Fluor ELWD 40× (NA, 0.6) objective and FRET module (GE Healthcare) (14, 61). Cells were sequentially excited using a CFP filter (430/24) with emission measured using YFP (535/30) and CFP (470/24) filters with a polychroic optimized for this filter pair (Quad 3). The FRET baseline was measured every 1 min for 4 min, before addition of vehicle control (0.0001% v/v MilliQ H_2_O) or 1 nM SP, with image capture continued for 20 min. At the end of each experiment, the same cells were stimulated with positive controls to maximally activate the biosensor: 200 nM phorbol 12,13-dibutyrate (PDBu) with phosphatase inhibitor cocktail (Merck) for CKAR, or 10 μM forskolin with 100 μM 3-isobutyl-1-methylxanthine for Epac2. After 10 min incubation, images were captured every 1 min for a final 4 min.

For fast confocal imaging experiments, HEK293 cells in 8-well Ibidi chamber slides were grown to 50% confluency before cotransfection with 110 ng/well HA-NK_1_R and 80 ng/well cytoCKAR. Before the experiment, cells were partially serum-restricted overnight in 0.5% (v/v) FBS DMEM. Forty-eight h after transfection, cells were preincubated with Span or Span-Chol (both 1 μM) for 4 h before the medium was replaced with HBSS and cells were equilibrated for 30 min at 37°C. Fast capture imaging was performed using a Zeiss LSM710 confocal fluorescence microscope with a Zeiss 40x NA1.34, oil immersion objective, with pinhole set to 2 AU. Cells were excited at 458 nm (CFP), with dual emission measured at 481 nm (CFP) and 540 nm (YFP). The FRET baseline was measured every 3 s for 30 s, before addition of vehicle control (0.0001% v/v MilliQ H_2_O) or 1 nM SP, with image capture continued every 3 s for 2 min. At the end of each experiment, the same cells were stimulated with a positive control, 200 nM PDBu, and imaged for a further 5 min.

For both high-content and fast imaging experiments, only cells with >3% change in F/F_0_ (FRET ratio relative to baseline for each cell) after stimulation with the positive controls were selected for analysis. The average F/F_0_ was calculated for each experiment and combined. Data were analyzed using in-house scripts written for the Fiji distribution of Image J (60), as described previously (61), with some modifications. The updated scripts are freely available from the Monash University online repository, Bridges (https://doi.org/10.26180/13289105) (62). Data were fit using a Pharmechanics “rise and fall” time course equation (“baseline then rise-and-fall to baseline time course with drift”), which is freely available (https://www.pharmechanics.com/time-course-tool-pack).

### Animal models of mechanical nociception

A total of 72 male C57Bl/6 mice (6–12 weeks old) were used in this study. Mice were maintained in a temperature and humidity-controlled room (23ºC ± 2º C) under a 12 h light/dark cycle with food and water *ad libitum*. The study was conducted in accordance with the requirements of the Australian Code for the Care and Use of Animals for Scientific Purposes (eighth edition, 2013) and the ethical guidelines of the International Association for the Study of Pain (63), and was approved by the animal ethics committee of Monash Institute of Pharmaceutical Sciences, Monash University. Mice were randomly assigned to experimental groups.

Mice were acclimatized to the experimental conditions on two successive days for 1–2 h. On the day of the study, withdrawal thresholds were measured in duplicate to establish baseline readings for each mouse. Span, Span-Chol, Chol (all 50 μM), or vehicle (1% v/v DMSO in 0.9% w/v saline) was injected intrathecally (5 μl, L3-L4) into the mice (n = 6 per group) anesthetized with isoflurane inhalation (2–5% delivered in oxygen). At 3, 6, and 12 h after drug administration, capsaicin (5 μg, vehicle: 20% ethanol, 10% Tween 80, 70% saline; v/v; 10 μl/mouse) was administered by intraplantar injection under isoflurane anesthesia (2–5% delivered in oxygen) to the left hindpaw. Nociception was assessed by measuring paw withdrawal thresholds with von Frey filaments of ascending force, applied to the plantar surface of the hindpaws as previously described (5, 64). Paw withdrawal thresholds were measured for both the ipsilateral and contralateral hindpaws every hour for 4 h. The data were subsequently normalized to the baseline paw withdrawal threshold for each animal. Investigators were blinded to drug treatments and experimental groups.

### Data analysis

Graphs were generated using GraphPad Prism 8 (San Diego, CA). Data are presented as mean ± S.E.M, unless otherwise stated.

## Data availability

All data are contained within the article.

## Conflict of interest

N. W. B. is a founding scientist of Endosome Therapeutics Inc. Research in the laboratories of N. W. B., N. A. V., and D. P. P. is funded in part by Takeda Pharmaceuticals Inc and Endosome Therapeutics Inc (N. A. V. and D. P. P.).
