# Supplementary material for: A lipid-anchored neurokinin 1 receptor antagonist prolongs pain relief by a three-pronged mechanism of action targeting the receptor at the plasma membrane and in endosomes
Source: J Biol Chem. 2021 Jan 28;296:100345. doi: 10.1016/j.jbc.2021.100345 (PMC7949131; doi:10.1016/j.jbc.2021.100345)
Supplement: Figures S1–S4 [file mmc1.pdf]

A lipid-anchored neurokinin 1 receptor antagonist prolongs pain relief by a three-pronged mechanism of action targeting the receptor at the plasma membrane and in endosomes

Quynh N. Mai[1,2,3,4], Priyank Shenoy[1,3], Tim Quach[2], Jeffri S. Retamal[2,3], Arisbel B. Gondin[1,3], Holly R. Yeatman[1], Luigi Aurelio[1,4], Joshua W. Conner[3,4], Daniel P. Poole[1,3], Meritxell Canals[5], Cameron J. Nowell[1], Bim Graham[4], Thomas P. Davis[2,3,6], Stephen J. Briddon[5], Stephen J. Hill[5], Christopher J. Porter[2,3], Nigel W. Bunnett[1,3,7,8,*], Michelle L. Halls[1,*], and Nicholas A. Veldhuis[1,2,3,*]

[1]Drug Discovery Biology Theme, [2]Drug Delivery, Disposition and Dynamics Theme, [3]Australian Research Council Centre of Excellence in Convergent Bio-Nano Science and Technology, and [4]Medicinal Chemistry Theme, Monash Institute of Pharmaceutical Sciences, Monash University, Parkville, VIC 3052, Australia;
[5]Division of Physiology, Pharmacology and Neuroscience, School of Life Sciences, The University of Nottingham Medical School, Nottingham, UK and Centre of Membrane Proteins and Receptors, Universities of Birmingham and Nottingham, the Midlands, U.K
[6]Australia Institute for Bioengineering and Nanotechnology, University of Queensland, Brisbane, QLD, 4072;
[7]Department of Pharmacology and Therapeutics, University of Melbourne, Parkville, VIC 3010, Australia
[8]Department of Molecular Pathobiology, New York University College of Dentistry, New York, NY 10010, USA.

QNM present address: Cardiovascular Research Institute, Department of Biochemistry and Biophysics, University of California, San Francisco, CA 94158

*Corresponding authors: Nicholas A. Veldhuis (nicholas.veldhuis@monash.edu), Michelle L. Halls (michelle.halls@monash.edu), Nigel W. Bunnett (nwb2@nyu.edu)

<u>List of Supporting Information</u>

Figure S1. Cartoon of NK_1R localized signaling
Figure S2. Structural cartoon of tripartite probes.
Figure S3. Addition of Chol alone has no effect on the NK_1R-induced recruitment of β-arrestin or receptor internalization to early endosomes.
Figure S4. Span inhibits NK_1R signaling from the plasma membrane, Span-Chol inhibits NK_1R signaling from endosomes.

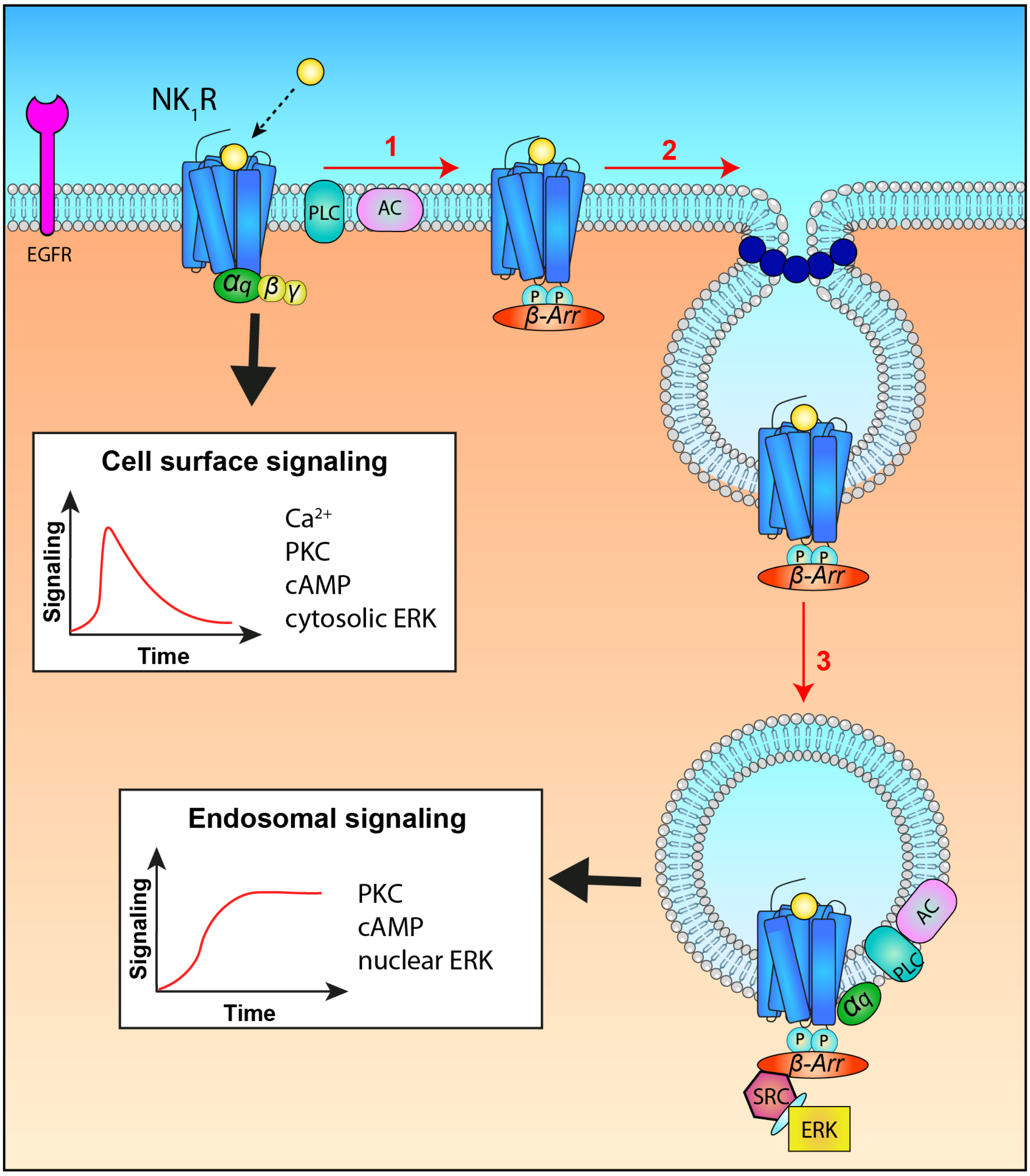

Figure S1. Cartoon of NK_1R localized signaling. Activation of the NK_1R causes two spatially and temporally distinct rounds of signaling. **1.** At the cell surface, SP-bound NK_1R rapidly activates $G\alpha_q$ G proteins to increase Ca^{2+} mobilization, protein kinase C (PKC) activity and cAMP in the vicinity of the plasma membrane (5,14). It also transactivates the epidermal growth factor receptor (EGFR) to stimulate extracellular signal-regulated kinase (ERK) activity in the cytoplasm. These signals are all relatively short-lived (<15 min) (14). **2.** During this time, GPCR kinases rapidly phosphorylate the NK_1R leading to association with β-arrestin and receptor internalization to early endosomes (<2 min) (5). **3.** Within endosomes, the SP-NK_1R complex continues to signal, causing increased PKC and cAMP in the cytosol and increased ERK within the nucleus (5,14). These signals from the endosomally localized receptor are longer-lived (>20 min). It is these sustained signals from the intracellular NK_1R that mediate the persistent excitation of spinal neurons and central pain transmission (5,7,14,15).

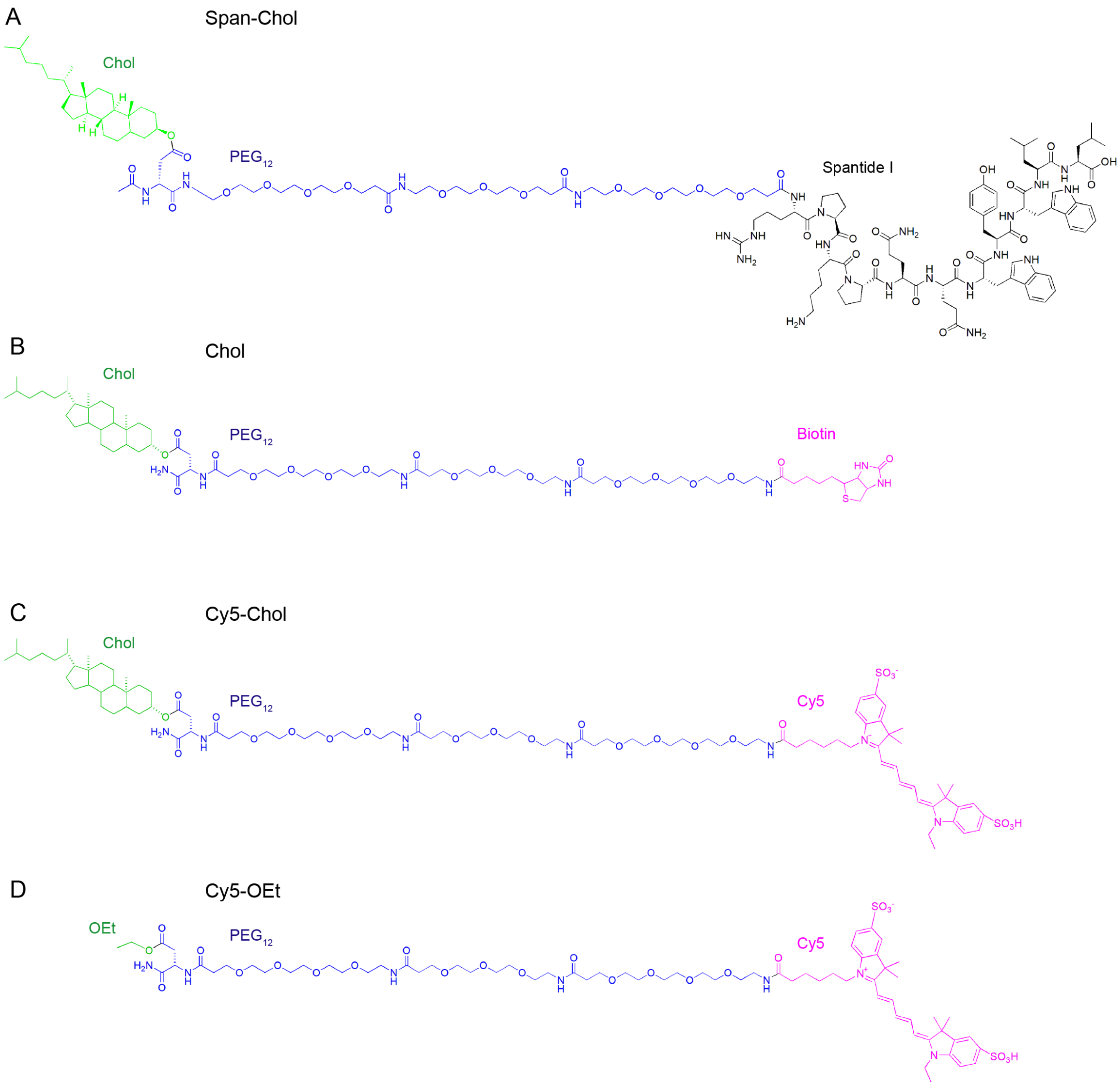

Figure S2. Structural cartoon of tripartite probes. The tripartite compounds used in this study consist of soluble cargo, conjugated to cholestanol or ethyl ester (non-lipidated control) via a polyethylene glycol (PEG) chain. **A.** Spantide I (black) conjugated via a PEG_{12} linker (blue) to cholestanol (green) to make Span-Chol. **B.** Biotin (magenta) conjugated via a PEG_{12} linker (blue) to cholestanol (green) to make Chol. **C.** Cy5 fluorescent reporter (magenta) conjugated via a PEG_{12} linker (blue) to cholestanol (green) to make Cy5-Chol. **D.** Cy5 fluorescent reporter (magenta) conjugated via a PEG_{12} linker (blue) to ethyl ester (green) to make Cy5-OEt.

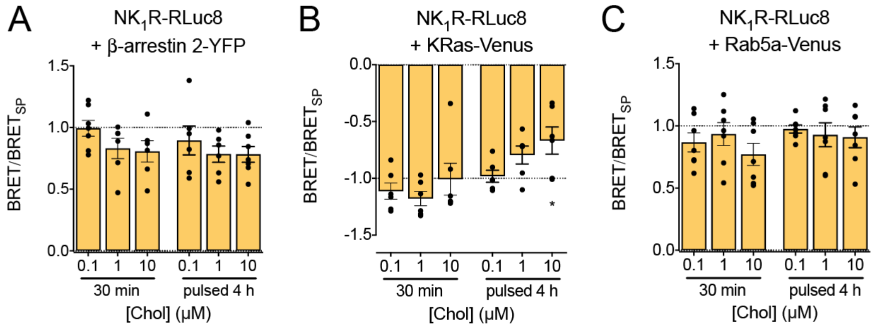

Figure S3. Addition of Chol alone has no effect on the NK$_1$R-induced recruitment of β-arrestin or receptor internalization to early endosomes. The effect of short (30 min) versus long (pulsed 4 h) pre-incubation with Chol on the NK$_1$R-induced recruitment of β-arrestin and receptor internalization to early endosomes was determined using BRET in HEK cells (n=5). **A.** 1 nM SP-induced change in BRET between NK$_1$R-Rluc8 and β-arrestin 2-YFP. **B.** 1 nM SP-induced change in BRET between NK$_1$R-Rluc8 and KRas-Venus. **C.** 1 nM SP-induced change in BRET between NK$_1$R-Rluc8 and Rab5a-Venus. Data are expressed as the plateau response calculated from curve fitting of the BRET time courses after pre-incubation with 0.1 μM, 1 μM or 10 μM antagonist, expressed relative to SP alone. Columns show means, error bars show S.E.M. and symbols show the mean of each individual experiment performed in duplicate.

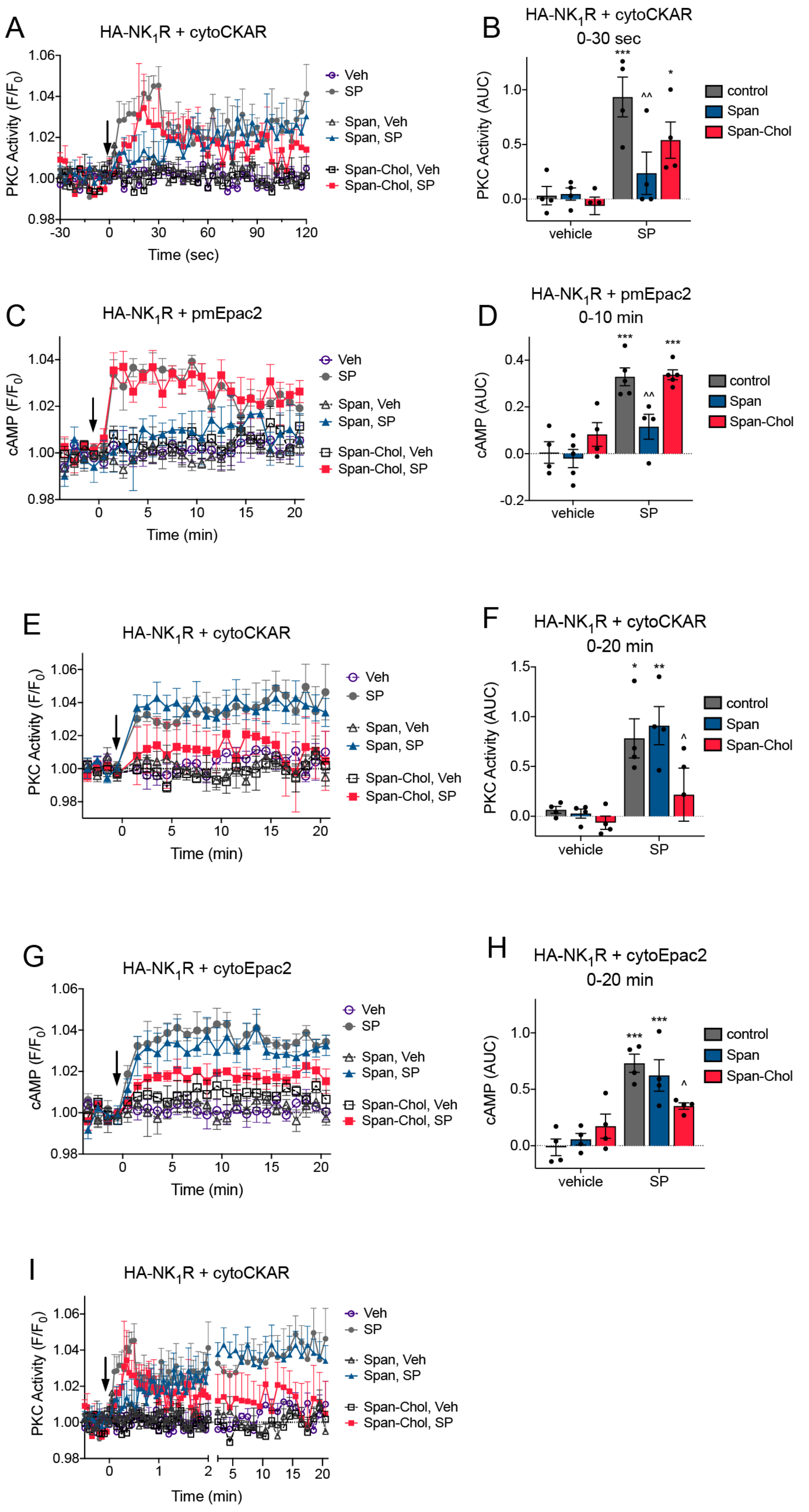
A
HA-NK1R + cytoCKAR
PKC Activity (F/F0)
Time (sec)
Veh
SP
Span, Veh
Span, SP
Span-Chol, Veh
Span-Chol, SP
B
HA-NK1R + cytoCKAR
0-30 sec
PKC Activity (AUC)
vehicle
SP
control
Span
Span-Chol
C
HA-NK1R + pmEpac2
cAMP (F/F0)
Time (min)
D
HA-NK1R + pmEpac2
0-10 min
cAMP (AUC)
E
HA-NK1R + cytoCKAR
PKC Activity (F/F0)
Time (min)
F
HA-NK1R + cytoCKAR
0-20 min
PKC Activity (AUC)
G
HA-NK1R + cytoEpac2
cAMP (F/F0)
Time (min)
H
HA-NK1R + cytoEpac2
0-20 min
cAMP (AUC)
I
HA-NK1R + cytoCKAR
PKC Activity (F/F0)
Time (min)

Figure S4. Span selective inhibits NK_1R signaling from the plasma membrane, whereas Span-Chol only inhibits NK_1R signaling from endosomes. Combined time courses (A,C,E,G) and calculated area under the curve (AUC; B,D,F,H) for Figures 5 and 6. **A.** Combined time courses from Figure 5A, 5D, 5F extended to 120 sec. **B.** 30 sec AUC calculated from A. **C.** Combined time courses from Figure 5B, 5E, 5G extended to 20 min. **D.** 10 min AUC calculated from C. **E.** Combined time courses from Figure 6A, 6D, 6F. **F.** 20 min AUC calculated from E. **G**. Combined time courses from Figure 6B, 6E, 6G. **H.** 20 min AUC calculated from G. **I.** Raw, non-smoothed data from Figure 6H. For time courses, data are expressed as the FRET relative to the baseline FRET (F/F_0), with symbols showing the mean, and error bars S.E.M. of grouped cells from 3-4 independent experiments. Arrows indicate time of vehicle/SP addition. For AUC graphs, bars show the mean, error bars the S.E.M. and symbols shows the averaged AUC from each independent experiment. * $p<0.05$, ** $p<0.01$ and *** $p<0.001$ versus vehicle control, two-way ANOVA with Sidak's multiple comparisons test. ^ $p<0.05$ and ^^ $p<0.01$ versus untreated control, two-way ANOVA with Dunnett's multiple comparisons test.
